# QALYs and rare diseases: exploring the responsiveness of SF-6D, EQ-5D-5L and AQoL-8D following genomic testing for childhood and adult-onset rare genetic conditions in Australia

**DOI:** 10.1186/s12955-023-02216-9

**Published:** 2023-12-12

**Authors:** Tianxin Pan, You Wu, James Buchanan, Ilias Goranitis

**Affiliations:** 1 Economics of Genomics and Precision Medicine Unit, Centre for Health Policy, Melbourne School of Population and Global Health, University of Melbourne, Parkville, Victoria Australia; 2 Australian Genomics Health Alliance, Melbourne, Victoria Australia; 3 Murdoch Children’s Research Institute, Melbourne, Victoria Australia; 4 Health Economics Research Centre, University of Oxford, Oxford, United Kingdom; 5 Health Economics and Policy Research Unit, Queen Mary University of London, London, United Kingdom

**Keywords:** Personal utility, Patient-reported outcome measures, Genomic sequencing, Rare disease, Responsiveness

## Abstract

**Background:**

Genomic testing transforms the diagnosis and management of rare conditions. However, uncertainty exists on how to best measure genomic outcomes for informing healthcare priorities. Using the HTA-preferred method should be the starting point to improve the evidence-base. This study explores the responsiveness of SF-6D, EQ-5D-5L and AQoL-8D following genomic testing across childhood and adult-onset genetic conditions.

**Method:**

Self-reported patient-reported outcomes (PRO) were obtained from: primary caregivers of children with suspected neurodevelopmental disorders (NDs) or genetic kidney diseases (GKDs) (carers’ own PRO), adults with suspected GKDs using SF-12v2; adults with suspected complex neurological disorders (CNDs) using EQ-5D-5L; and adults with dilated cardiomyopathy (DCM) using AQol-8D. Responsiveness was assessed using the standardised response mean effect-size based on diagnostic (having a confirmed genomic diagnosis), personal (usefulness of genomic information to individuals or families), and clinical (clinical usefulness of genomic information) utility anchors.

**Results:**

In total, 254 people completed PRO measures before genomic testing and after receiving results. For diagnostic utility, a nearly moderate positive effect size was identified by the AQoL-8D in adult DCM patients. Declines in physical health domains masked any improvements in mental or psychosocial domains in parents of children affected by NDs and adult CNDs and DCM patients with confirmed diagnosis. However, the magnitude of the changes was small and we did not find statistically significant evidence of these changes. No other responsiveness evidence related to diagnostic, clinical, and personal utility of genomic testing was identified.

**Conclusion:**

Generic PRO measures may lack responsiveness to the diagnostic, clinical and personal outcomes of genomics, but further research is needed to establish their measurement properties and relevant evaluative space in the context of rare conditions. Expected declines in the physical health of people experiencing rare conditions may further challenge the conventional application of quality of life assessments.

**Supplementary Information:**

The online version contains supplementary material available at 10.1186/s12955-023-02216-9.

## Key points

While a growing body of literature has examined the validity of generic and disease-specific patient-reported outcome measures (PROMs) in rare conditions and discussed the challenges of using PROMs in the context of genomic medicine, there is limited empirical evidence exists to demonstrate the responsiveness of generic PROMs in the context of genomic testing.

We aimed to provide empirical evidence on the responsiveness of three common generic PROMs (SF-6D, EQ-5D-5L and AQoL-8D) in terms of the presence of diagnostic, personal, and clinical utility of genomics, by using data from four cohorts recruited within the Australian and Melbourne Genomics Health Alliances, 2016–2019.

Generic PROMs may lack responsiveness to the diagnostic, clinical and personal outcomes of genomics. Expected declines in the physical health of people experiencing rare conditions may further challenge the conventional qapplication of uality of life assessments.

**Supplementary Information:**

The online version contains supplementary material available at 10.1186/s12955-023-02216-9.

## Introduction

There are 6,000–8,000 known rare diseases affecting 263–446 million people globally, with 80% of rare diseases having a genetic cause [1]. Rare diseases can be life-threatening and/or debilitating, and patients often have long journeys to diagnosis involving many tests and visits to hospital specialists. Improved knowledge and technological advances in gene sequencing are transforming the diagnostic trajectory of rare genetic conditions, and a growing body of evidence highlights the potential diagnostic, clinical and personal utility (i.e. the non-health outcomes relates to the personal rationales for and benefit derived from genomic technologies, regardless of its potential to improve health [2–4]) and economic benefits of genomic testing [5–9]. These outcomes are valued highly by society and those experiencing genetic conditions [10–12]. The implementation of genomics into mainstream clinical care, however, is falling behind technological and research advances [13]. One potential explanation for the slow mainstreaming of genomics is that next generation sequencing is a complex technology inherently different from conventional targeted diagnostic tests and other health technologies that are commonly assessed within established Health Technology Assessment (HTA) evaluation frameworks [3, 14].

HTA agencies commonly make reimbursement recommendations on the basis of health economic evidence generated using cost-utility analyses, whereby Quality-Adjusted Life-Years (QALYs) represent the standard unit of outcome [15–17]. QALYs are a composite measure of length of life and quality of life. The quality adjustment is widely implemented using generic patient-reported outcome measures (PROMs) accompanied by preference weights, such as the EQ-5D [18] and SF-6D [19]. These instruments were designed to measure a core set of domains that are linked to health and provide a means to compare across diseases or changes in health. However, genomic testing has the potential to generate a wide range of health and non-health outcomes which have implications for individuals and families. Such outcomes are challenging to measure and QALYs do not incorporate preferences for non-health outcomes.

While a growing body of literature has examined the validity of generic and disease-specific PROMs in rare diseases [20–22] and discussed the challenges of using PROMs in the context of genomic medicine [14], there is limited empirical evidence available to demonstrate the responsiveness of generic PROMs in rare diseases, particularly genomic testing [23, 24]. A recent review of HTA appraisals of non-cancer European Medicines Agency orphan medicinal products in the UK, the Netherlands, France and Germany found between 16% and 61% of the included appraisals did not report patient-reported outcome evidence, and when reported, the results (e.g. impact of treatments on outcomes) were not discussed [25]. This lack of evidence has also been explicitly recognized by HTA agencies (e.g. Medical Services Advisory Committee in Australia, MSAC) [26] and instrument developers (e.g. EuroQol Group) which called for further evidence on the performance of EQ-5D instruments in rare diseases [27]. This study aims to provide empirical evidence on the responsiveness of three common generic PROMs (SF-6D, EQ-5D-5L and AQoL-8D) in terms of the presence of diagnostic, personal and clinical utility of genomic testing, by using data from four cohorts recruited within the Australian and Melbourne Genomics Health Alliances, 2016–2019.

## Methods

This study used existing clinical and patient-reported outcome (PRO) data collected prospectively through the Australian Genomics and Melbourne Genomics Health Alliances programs. More specifically, we use self-reported HRQoL data from: (a) parents of children affected by neurodevelopmental disorders (NDs), including mitochondrial disorders, developmental epileptic encephalopathy, and brain malformations, (b) adult patients and parents of children affected by genetic kidney diseases (GKDs), (c) adult patients with complex neurological and neurodegenerative disorders (CNDs), (d) adults with dilated cardiomyopathy (DCM). More information on these cohorts is provided in Appendix A.

### Study design and participants

#### Neurodevelopmental disorders (NDs)

Families with a child suspected with mitochondrial disorder, epileptic encephalopathy, or brain malformation were prospectively recruited across all the states except for Australian Capital Territory in Australia, 2017–2019 to assess the diagnostic yield of exome sequencing in children with suspected NDs [28, 29]. Parental health-related quality of life (HRQoL) was assessed using the second version of the SF-12 Health Survey (SF-12v2) at recruitment and 3 months post-test results disclosure, which approximately corresponds to a 7-month period.

The **SF-12v2** is a generic PROM, consisting of a subset of 12 items from the SF-36 Health Survey (SF-36v2) [30], covering physical functioning, role limitations due to physical functioning (role physical), bodily pain, general health, vitality, social functioning, role limitations due to emotional functioning (role emotional), and mental health [31]. Summary scales can be computed to reflect physical well-being (the Physical Component Summary, PCS) and mental well-being (Mental Component Summary, MCS).

We calculated PCS and MCS scores following the scoring manual, which were standardized to have a mean of 50 and a standard deviation (SD) of 10 similar to the US general population [31, 32]. To enable the use of the SF-36 and SF-12 instruments in economic evaluation, a concatenated version (SF-6D) has been developed based on stated preferences from the general population. This covers six domains in the SF-12 instrument, excluding the general health item and combining two role limitation domains. More recently, a newer version of SF-6D (SF-6Dv2) was developed [33] and a value set has been developed in Australia [34]. However, the descriptive system of SF-6Dv2 was not restricted to SF-12v2 items thus SF-6Dv2 utility scores cannot be directly applied from SF-12 [33, 35]. The value set for SF-12v2 or the algorithm to map SF-12v2 response to SF-6Dv2 utilities is not available, therefore, we calculated the utility scores using the preference weights generated for SF-12 from the UK general population [31].

#### Genetic kidney diseases (GKDs)

A national study was conducted across multiple states in Australia (New South Wales, Queensland, South Australia, Victoria, Western Australia) to evaluate the diagnostic yield of genomic testing for the following childhood- and adult-onset GKDs, 2017–2019: Alport syndrome, nephrotic syndrome, other glomerular disease, cystic kidney disease, tubular disease, complement disorder, congenital renal disease and others [28, 36–38]. The study assessed parental (for childhood-onset conditions) and adult patients’ quality of life at recruitment and after receiving the test results using the SF-12v2. Quality of life data from the SF-12v2 were analyzed as described in the section on NDs above.

#### Complex neurological and neurodegenerative diseases (CNDs)

A prospective multi-site study in the State of Victoria, Australia was conducted between August 2017 and October 2018 to assess the diagnostic yield of exome sequencing in patients with the following neurological phenotypes of suspected genetic aetiology: ataxia, dementia, spastic paraplegia, dystonia, motor neuron disease, Parkinson’s disease, and complex/unspecified neurological disease [9]. Adult patients recruited in the study were asked to complete the EQ-5D-5L, Depression, Anxiety and Stress Scale (DASS) 21 item, and Neurology-Quality of Life (Neuro-QoL) Positive Affect and Well-being (PAW) measure at recruitment and 2–3 weeks after receiving the test results.

The **EQ-5D-5L** is a widely used generic outcome measure accompanied by preference weights. It includes five dimensions: mobility, self-care, usual activities, pain/discomfort, anxiety/depression, with each dimension having five response levels ranging from 1 (no problems) to 5 (unable/extreme problems). In this study, we used the value set developed for Australia [39]. In a sensitivity analysis, we used the value set developed for England and US respectively [40, 41]. Participant responses to the EQ visual analogue scale (EQ VAS), which records their subjective assessment of their own health, were rescaled to a 0 (worst imaginable health) to 1 (best imaginable heath) scale.

The **21-item DASS** (depression, anxiety, stress scale) evaluates these three dimensions, with a 7-item scale in each dimension. Z-scores were calculated for each dimension following the scoring manual [42]. To enable an intuitive interpretation of DASS scores in relation to the other measures in the study, scores were rescaled so that higher scores reflected better health states.

The **Neuro-QoL PAW** is a 9-item short form survey reflecting components of positive affect, life satisfaction, or an overall sense of purpose and meaning [43]. We transformed PAW raw scores to T-scores, which are standardized scores with a mean of 50 and SD of 10 for the general US population [44]. A higher T-score represents better neurology-related quality of life [43].

#### Dilated cardiomyopathy (DCM)

Adults with idiopathic DCM or other non-hypertrophic cardiomyopathies (e.g., restrictive cardiomyopathies) were recruited for genomic testing in the State of Victoria, Australia between April 2016 and September 2017 [45, 46]. The study assessed quality of life using the Assessment Quality of Life 8D measure (AQoL-8D) at recruitment and 2–3 weeks post-results disclosure.

The **AQoL-8D** is a validated measure that contains 35 items and comprises eight separately scored domains, consisting of independent living, relationships, mental health, coping, pain, senses, self-worth and life satisfaction [47]. Three of these domains (independent living, pain and senses) are related to a ‘Physical’ overarching dimension and the remaining five to a ‘Psychosocial’ dimension. The utility algorithm used in this study was derived from the Australian general population [48, 49].

### Responsiveness analysis

The responsiveness of generic PROMs reflects their ability to capture meaningful changes in health and well-being [50]. We assessed responsiveness across 3 anchors that are known to be of high importance to adult patients and parents of children experiencing rare disease [51]. These anchors are: (a) diagnostic utility, defined as receiving a genetic diagnosis (Analysis 1); (b) personal utility, which reflects the value of different non-health and non-clinical outcomes of genomic testing to individuals from their own personal perspective (Analysis 2); and (c) clinical utility, defined as the presence of clinically meaningful changes in patient care on the basis of genomic information (Analysis 3). As recommended [52], responsiveness was evaluated using the Standardised Response Mean (SRM) effect size statistic, calculated as the ratio of the mean change of utility scores to the SD of the changes in scores. The effect size indicates the relative size of the ‘signal’ in comparison with underlying ‘noise’ in the data. Studies have highlighted the importance of reporting effect size (substantive significance) in addition to p-value (statistical significance) [53]. A positive effect size reflects positive changes in utility scores (increase in health utilities) while a negative effect size reflects declines in health utilities. The values 0.2, 0.5, and 0.8 were used as thresholds for small, moderate, and large effect sizes [54]. Paired *t*-test and Wilcoxon’s test were carried out to identify statistically significant changes in scores at the 95% confidence level. Studies have established the importance of assessing responsiveness and reporting effect size [50, 53]. Several sensitivity analyses were performed by using different preference weights, reporting changes in health at a dimension level, and health states without introducing preference weights. We further explored patterns of changes in health states among those who had increased health utilities. Within each of the analyses described below we present results separately for parents of affected children and adult patients. Given the differences between clinical studies included in our analyses and their objectives, all cohorts contributed to Analyses 1 and 2, with the NDs group contributing to Analysis 3.

#### Analysis 1: Assessment of responsiveness based on diagnostic utility

In this analysis, changes in health scores (including utility scores, domain scores and/or component summary scores) across PROMs were evaluated, and responsiveness was assessed, for two groups: those who received a molecular diagnosis and those who did not. All patients in the cohorts received an outcome from genomic testing. The diagnostic rate in each cohort was 32% (NDs parents), 50% and 53% (GKDs parents and adults), 16% (CNDs adults) and 18% (DCM adults) [9, 37, 46, 55]. Analysis 1 was conducted among those who responded to baseline and follow up surveys and had completed information on PROMs. The baseline survey was conducted after attending genetics clinics at which patients were offered and consent to testing (or 2–3 weeks after attending genetics clinics). The follow up survey was conducted following the receipt of genomic test results (or 2–3 weeks after receiving the test results). The duration for generating results and yield diagnosis may vary between 3 and 6 months depending on the conditions and individual variations. Therefore, the duration between the two surveys is approximately 7 months.

#### Analysis 2: Assessment of responsiveness based on personal utility

All the included studies collected information on how valuable genomic testing had been for the participants and their family, in the follow up survey after participants receiving the test results. The following statements were presented and respondents were asked to answer on a 4-level scale including “extremely valuable”, “valuable”, “neutral”, “not valuable”, and “not applicable” (if the test has not provided the described impact to the question): (1) ongoing investigations no longer necessary; (2) knowing the cause/explanation for the condition; (3) receiving information for family planning; (4) receiving information for other family members; (5) informing treatment/management of the condition; (6) providing information on prognosis; (7) having had access to the most recent advances in medicine; (8) have done everything I can to improve health; (9) ability to connecting with others with the same condition. In addition, NDs patients were asked another statement: (10) contributing to research. DCM patients were asked (10) and (11) having data be examined in more detail to find an answer. These statements cover different aspects of potential value of genomic testing.

To facilitate assessment of responsiveness, we used factor analysis to construct an indicator of personal utility using the responses to these statements. The factor analysis was conducted among those who responded to these statements, for each study independently. Among parents of children affected by NDs, the factor ‘providing information’ was identified and used to construct a binary indicator for personal utility. In the CNDs, GKDs and DCM groups, two main factors were identified to construct two binary personal utility indicators; one reflecting the value in providing information on causes, treatment, prognosis and family planning, and another reflecting value in maximizing chances to improve health. The proportion of respondents found value in providing information to those who responded to perceived value questions was 75% (NDs parents), 92% and 82% (GKD parents and adult patients), 66% (CNDs adult patients) and 60% (DCM adult patients); the proportion of respondents found value in maximizing chances to improve health to those who responded to perceived value questions was 95% and 81% (GKD parents and adult patients), 77% (CNDs adult patients) and 77% (DCM adult patients). Additional information on constructing the personal utility indicator is provided in Appendix B. Analysis 2 was conducted among those who had complete information on PROs in both waves and responded to personal utility statements. For the two constructed indicators, changes in PROs were assessed between people who found value and those who did not, respectively. In a sensitivity analysis, for each of the 11 statements mentioned above, we reported changes in PROs among those who found value in genomic testing and those who did not.

#### Analysis 3: Assessment of responsiveness based on clinical utility

Clinical utility in the NDs cohort was derived from two clinician-reported surveys. We used clinician responses to the questions *“Whether genomic testing was useful in changing management”* and *“Whether genomic testing was useful in improving health”* to construct two binary indicators for clinical utility. There were four responses to each question: not useful, neutral, useful, and very useful. A value of 1 was given to the clinical utility indicator if ‘useful’ or ‘very useful’ were chosen and 0 otherwise. Responsiveness was assessed for two groups: participants whose test results were classed as clinically useful (59%), and those whose results were not classed as clinically useful (41%).

All statistical analyses were conducted using STATA 15.1 SE, and p-values of less than 0.05 were considered statistically significant.

## Results

### Sample characteristics

In total, 254 people responded to both baseline and follow-up surveys and had complete data on PROMs from the four cohorts, with 33% representing parents of children living with rare disease and 67% representing adult patients affected by rare disease. In the NDs study (total n = 273 children), 62 (22%) of their primary caregivers completed the SF-12v2 at both baseline and follow-up. In the GKDs study (total n = 115 children and n = 252 adult patients), 22 primary caregivers (19%) and 76 (30%) adult patients responded to the baseline and follow-up SF-12v2, respectively. In the CNDs study (total n = 154 adult patients), 61 (40%) responded to the EQ-5D-5L in both periods. In the DCM study (total n = 62 adult patients), 33 (53%) completed the AQol-8D at baseline and follow-up. The sample flow chart and the characteristics of each cohort in our analytical sample (and the characteristics of participants who were recruited in each cohort) are reported in Appendix C. Mean baseline health utilities for parents of children with suspected NDs, GKDs, adults with suspected GKDs, adult CNDs patients and adult DCM patients were 0.72, 0.70, 0.72, 0.71 and 0.71 respectively. These health utilities were measured by different PROMs as reported in the Methods section, thus may not be directly comparable.

### Main results

#### Analysis 1: Assessment of responsiveness based on diagnostic utility

**Fig. 1:**
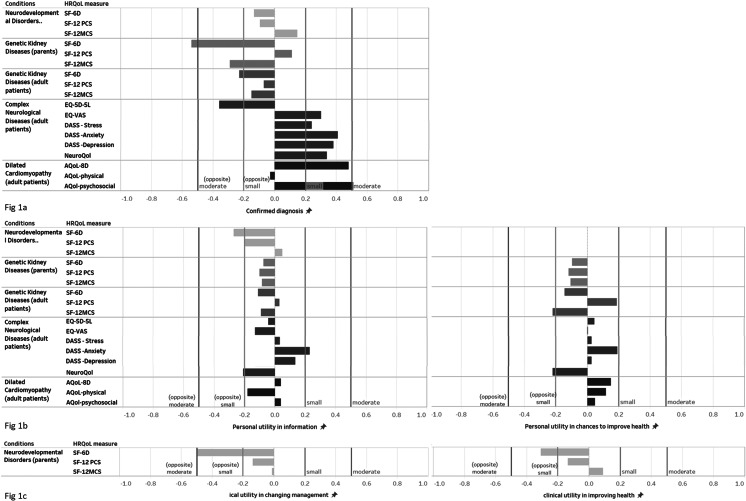
Responsiveness of patient-reported outcome measures to genomic testing in rare disease. (**a**) Responsiveness of PROMs to diagnostic utility. (**b**) Responsiveness of PROMs to personal utility. (**c**) Responsiveness of PROMs to clinical utility. Responsiveness was evaluated using the Standardised Response Mean (SRM) effect size statistic, calculated as the ratio of the mean change of index scores to the SD of the changes in scores. The values 0.2, 0.5, and 0.8 were used as thresholds for small, moderate, and large effect sizes. The red, pink, orange and green reference lines refer to negative moderate effect (-0.5), negative small effect (-0.2), positive small effect (0.2) and positive moderate effect (0.5) respectively

Table 1 shows the mean changes in utility scores and domain component scores between baseline and follow-up across conditions depending on whether a confirmed molecular diagnosis was received or not. Among parents of children affected by NDs, there was, on average, a small decline in SF-6D utilities (=-0.01) among parents whose child received a diagnosis. There was also a small decline in the physical health component (=-1.05) while an increase in the mental health component (= 1.97). However, these changes were not statistically significant and no relevant effect sizes were identified for parents whose child received a genetic diagnosis (Fig. 1a). Among parents whose child did not receive a diagnosis, we did not find statistically significant decline in mean utility scores at the 5% level (while significant at 10% level). No differences were identified in the parental physical or mental health component depending on whether the child was diagnosed or not, at 5% significance level.

**Table 1:** Responsiveness of PROMs between baseline and follow-up of parents or patients with different rare diseases by diagnostic outcome

	All					Diagnosis					No diagnosis				
		Baseline		Follow up		Change					Change				
	N	Mean	SD	Mean	SD	N	Mean	SD	*p*-value	SRM	N	Mean	SD	*p*-value	SRM
NDs (parents)															
SF-6D	62	0.72	0.12	0.69	0.13	20	-0.01	0.08	0.56	-0.13	42	-0.04	0.14	0.06*	-0.29a
SF-12 PCS	62	53.36	8.35	50.89	9.95	20	-1.05	10.81	0.67	-0.10	42	-3.15	10.25	0.053*	-0.31a
SF-12 MCS	62	42.43	12.61	42.55	10.75	20	1.97	13.44	0.52	0.15	42	-0.76	14.80	0.74	-0.05
GKDs (parents)															
SF-6D	22	0.70	0.12	0.69	0.11	11	-0.04	0.07	0.10	-0.54b	11	0.01	0.11	0.66	0.14
SF-12 PCS	22	51.71	9.39	51.67	9.92	11	0.60	5.43	0.72	0.11	11	-0.69	8.17	0.79	-0.08
SF-12 MCS	22	40.84	12.42	39.44	12.83	11	-2.79	9.47	0.35	-0.29a	11	0.00	9.14	0.999	0.00
GKDs (adult patients)															
SF-6D	76	0.72	0.13	0.71	0.14	40	-0.02	0.09	0.16	-0.23a	36	0.01	0.08	0.36	0.15
SF-12 PCS	76	47.92	11.74	49.35	10.72	40	-0.54	7.57	0.66	-0.07	34	3.73	9.32	0.03 **	0.40a
SF-12 MCS	76	44.97	11.81	43.49	12.52	40	-1.25	8.38	0.35	-0.15	34	-1.74	9.54	0.30	-0.18
CNDs (adult patients)															
EQ-5D-5L															
Utility score	61	0.71	0.29	0.70	0.28	10	-0.06	0.16	0.29	-0.35a	51	0.01	0.12	0.73	0.05
EQ VAS score	59	0.67	0.20	0.66	0.21	9	0.05	0.18	0.40	0.30a	50	-0.02	0.17	0.50	-0.12
DASS															
Depression	52	-0.42	1.58	-0.4	1.48	9	0.48	1.26	0.33	0.38a	43	-0.08	1.23	0.17	-0.06
Anxiety	52	-0.73	1.78	-0.55	1.71	9	0.63	1.57	0.33	0.41a	43	0.09	0.98	0.48	0.09
Stress	52	-0.19	1.36	-0.15	1.24	9	0.25	1.04	0.51	0.24a	43	0.00	1.00	0.93	0
Neuro QoL															
PAW	55	53.08	7.48	52.69	8.36	9	1.00	2.96	0.34	0.34a	46	-0.66	5.74	0.44	-0.12
DCM (adult patients)															
AQol-8D	33	0.71	0.18	0.72	0.17	6	0.04	0.07	0.29	0.48a	27	0.01	0.11	0.77	0.06
Physical	33	0.66	0.21	0.66	0.22	6	-0.002	0.08	0.95	-0.03	27	0.001	0.13	0.96	0.01
Psychosocial	33	0.40	0.17	0.42	0.17	6	0.04	0.08	0.26	0.51b	27	0.01	0.12	0.72	0.07

*Note*: PROMs: patient-reported outcome measures; ND: neurodevelopmental disorders; GKD: genetic kidney diseases; CNDs: complex neurological disorders; DCM: dilated cardiomyopathy. For ND parents, GKD parents and GKD adults, a higher SF-6D score, SF-12 Physical Component Scores (PCS) and SF-12 Mental Component Scores (MCS) indicate better health. t-test was used to assess the mean change in SF-6D score, SF-12 PCS score and SF-12 MCS score. For CND adult patients, a higher EQ-5D utility score and EQ VAS score indicates better health. We rescaled EQ VAS score to a 0–1 scale; DASS scale score was converted to z-score to enable comparison between DASS scale scores. We rescaled the DASS scores so that higher z-score indicates better health state (i.e. 0- original z-score). A higher Neuro-QoL T-score represents more of the concept being measured. t-tests was used to assess the mean change in EQ-5D utility score, EQ VAS score and NeuroQoL score and Wilcoxon’s test was used to assess the mean change in DASS score. For adult DCM patients, a higher AQoL utility score and the two component scores indicate better health. t-test was used to assess the mean change in AQoL utility score. The values of 0.2, 0.5 and 0.8 represent the cut-off points for small (a), moderate (b), and large (c) standardised response mean (SRM) effect sizes *, **, *** denote significance at the 10, 5, and 1% level, respectively

Among parents of children affected by GKDs, there was on average a 0.04 decline in SF-6D utilities among parents whose child received a molecular diagnosis. The negative effect size was moderate (=-0.54) but not statistically significant at 5% level. The decline was mostly related to parental mental health (mean change=-2.79) rather than physical health (mean change = 0.60). The opposite pattern was observed among parents whose child did not receive a diagnosis. For adult GKDs patients who received a molecular diagnosis, reductions in SF-6D utilities and PCS and MCS scores were observed, but we did not find statistical significance of these changes. In adult GKDs patients who did not receive a diagnosis, there was a 0.01 increase in health utility, largely driven by the increase in PCS score (mean change = 3.73, with a small effect size of 0.40, statistically significant at 5% level).

For adult patients with a confirmed CND diagnosis there was on average a decline in health-related quality of life, as evidenced by decreased EQ-5D-5L utility scores (=-0.06) and a small negative effect size (=-0.35). However, small positive effect sizes were identified for the EQ VAS (mean change = 0.05, effect size = 0.30), three domains of DASS and PAW, which reflect improvements in overall self-assessed health (that goes beyond the five dimensions described in EQ-5D) and mental health domains, respectively.

Among adult DCM patients, nearly moderate effect sizes (= 0.48) were identified for those who received a confirmed molecular diagnosis. These effect sizes were driven by the psychosocial domains of the measure (mean change = 0.04, effect size = 0.51) rather than physical domains (mean change = -0.002, effect size = -0.03).

Declines in physical health domains masked any improvements in mental or psychosocial domains, in parents of children affected by NDs, adult CNDs patients and DCM patients with confirmed diagnosis. It is noteworthy that the standard deviations of the estimated mean change in health utility in each sample were high, indicating heterogeneity in the changes in PROs within each cohort. We performed sensitivity analyses and additional analyses to explore changes in PROs among those who experienced increased health utilities and declined health utilities, respectively. Among those who had increased health utilities, between 74 and 95% parents or adult patients report improvements on at least one mental health related domain (the Anxiety/depression dimension for EQ-5D-5L in adult CND patients; the Role limitation, Social functioning, Vitality, and Mental health domains for SF-6D in parents of children with NDs or GKDs and adult GKDs patients; the Happiness, Relationship and Self-worth domains for AQoL-8D in adult DCM patients). Even among those who experienced declined utilities, we still observed between 26 and 64% of them reported improvements on mental or psychosocial related domains (while having reported decline in physical health related domains). Detailed analysis and results are reported in Appendix D-G.

#### Analysis 2: Assessment of responsiveness based on personal utility

Table 2 reports changes in PROs depending on the presence of personal utility derived from genomic testing across the four conditions. For the parents of children affected by NDs, small negative effect sizes were identified, indicating declines in outcomes, regardless of whether parents perceived that genomic testing yielded personal utility. For parents of children with GKD and adult patients with GKDs, CNDs or DCM who found value in genomic testing in terms of providing information, no relevant effect sizes on changes in health utilities were identified (Fig. 1b). Similarly, no relevant effect sizes were identified among those who found value in terms of maximizing chances to improve health (Fig. 1b). The pattern was similar when we examined the change in outcomes by each of the 11 items (as described in Method section) that we used to construct the personal utility indicator, and by level of constructed indicator (results presented in Appendix H).

**Table 2:** Responsiveness of PROMs of parents or patients with different rare diseases by perceived value of diagnostic genomic sequencing

	Value of genomic sequencing in providing information										Value in maximizing chance to improve health									
	Among those who found value in this aspect					Among those who did not found value					Among those who found value in this aspect					Among those who did not found value				
Changes in PROs	N	Mean	SD	*p-*value	SRM	N	Mean	SD	*p-*value	SRM	N	Mean	SD	*p-*value	SRM	N	Mean	SD	*p-*value	SRM
NDs (parents)																				
SF-6D	44	-0.03	0.12	0.08	-0.27a	13	-0.04	0.13	0.23	-0.35a										
SF-12 PCS	44	-2.18	10.66	0.18	-0.20a	13	-2.24	8.25	0.35	-0.27a										
SF-12 MCS	44	0.73	13.27	0.72	0.05	13	-5.26	14.63	0.22	-0.36a										
GKDs (parents)																				
SF-6D	17	-0.01	0.10	0.77	-0.07	2	0.00	0.06	0.95	0.05	18	-0.01	0.10	0.69	-0.10	1	0.05	NA	NA	NA
SF-12 PCS	17	-0.74	7.23	0.68	-0.10	2	1.61	5.74	0.76	0.28a	18	-0.84	7.03	0.62	-0.12	1	5.66	NA	NA	NA
SF-12 MCS	17	-0.81	9.57	0.73	-0.09	2	0.22	5.70	0.96	0.04	18	-0.98	9.31	0.66	-0.11	1	4.25	NA	NA	NA
GKDs (adult patients)																				
SF-6D	55	-0.01	0.09	0.42	-0.11	12	-0.01	0.10	0.80	-0.08	55	-0.01	0.09	0.29	-0.14	12	0.00	0.12	0.93	0.03
SF-12 PCS	54	0.24	8.61	0.84	0.03	12	2.32	6.93	0.27	0.33a	54	1.44	7.71	0.18	0.19	12	-3.06	10.20	0.32	-0.30
SF-12 MCS	54	-0.73	7.95	0.50	-0.09	12	-0.78	9.97	0.79	-0.08	54	-1.69	7.63	0.11	-0.22a	12	3.52	9.97	0.25	0.35a
CNDs (adult patients)																				
EQ-5D-5L																				
utility score	37	-0.01	0.15	0.76	-0.05	17	0.01	0.12	0.70	0.09	42	0.01	0.15	0.78	0.04	12	-0.03	0.07	0.39	-0.43a
EQ VAS score	35	-0.02	0.19	0.46	-0.13	17	0.03	0.14	0.45	0.19	40	0	0.19	0.98	0	12	-0.04	0.13	0.39	-0.26a
DASS																				
Depression	30	0.21	1.55	0.78	0.14	15	-0.33	0.65	0.1	-0.51b	33	0.04	1.47	0.42	0.03	12	0	0.89	1	0.00
Anxiety	30	0.29	1.26	0.32	0.23a	15	-0.03	0.99	0.98	-0.03	33	0.24	1.25	0.43	0.19	12	0.04	0.97	0.64	0.04
Stress	30	0.04	1.22	0.98	0.03	15	0.2	0.52	0.11	0.38a	33	0.03	1.16	1	0.03	12	0.27	0.61	0.12	0.44a
Neuro QoL																				
PAW	35	-1.12	5.42	0.23	-0.21a	15	-0.19	4.79	0.88	-0.04	39	-1.18	5.28	0.17	-0.22a	11	0.35	5.01	0.82	0.07
Dilated cardiomyopathy (adult patients)																				
AQoL-8D	17	0	0.1	0.88	0.04	11	0.02	0.11	0.54	0.19	23	0.01	0.09	0.48	0.15	5	0	0.16	0.97	-0.02
Physical	17	-0.02	0.11	0.46	-0.18	11	0.04	0.16	0.4	0.26a	23	0.01	0.13	0.58	0.12	5	-0.05	0.16	0.53	-0.30a
Psychosocial	17	0	0.1	0.86	0.04	11	0.01	0.09	0.68	0.13	23	0	0.09	0.81	0.05	5	0.02	0.11	0.72	0.17

*Note*: PROMs: patient-reported outcome measures; ND: neurodevelopmental disorders; GKD: genetic kidney diseases; CNDs: complex neurological disorders; DCM: dilated cardiomyopathy. For ND parents, GKD parents and GKD adults, a higher SF-6D score, SF-12 Physical Component Scores (PCS) and SF-12 Mental Component Scores (MCS) indicate better health. t-test was used to assess the mean change in SF-6D score, SF-12 PCS score and SF-12 MCS score. For adult CND patients, a higher EQ-5D utility score and EQ VAS score indicates better health. We rescaled EQ VAS score to a 0–1 scale; DASS scale score was converted to z-score to enable comparison between DASS scale scores. We rescaled the DASS scores so that higher z-score indicates better health state (i.e. the rescale score = 0- original z-score). A higher Neuro-QoL T-score represents more of the concept being measured. t-tests was used to assess the mean change in EQ-5D utility score, EQ VAS score and NeuroQoL score and Wilcoxon’s test was used to assess the mean change in DASS score. For adult DCM patients, a higher AQoL utility score and the two component scores indicate better health. t-test was used to assess the mean change in AQoL utility score.

The values of 0.2, 0.5 and 0.8 represent the cut-off points for small (a), moderate (b), and large (c) standardised response mean (SRM) effect sizes

*, **, *** denote significance at the 10, 5, and 1% level, respectively

#### Analysis 3: Assessment of responsiveness based on clinical utility

We examined the responsiveness of the SF-6D based on the presence of clinical utility following genomic testing among parents of children with NDs (Table 3). Where genomic testing was useful in changing the child’s clinical management, moderate negative effect sizes were identified that indicated a decline in mean utility scores for parents (Fig. 1c). Small negative effect sizes were also identified in parents regardless of whether genomic testing led to improvements in the child’s health based on the clinician’s response (Fig. 1c).

**Table 3:** Responsiveness of PROMs of based on clinical utility among parents of children affected by ND

	Genomic testing useful in changing management												Genomic testing useful in improving health										
	Yes						No						Yes						No				
Changes in HRQoL	N	Mean	SD	p-value	SRM		N	Mean	SD	p-value	SRM		N	Mean	SD	p-value	SRM		N	Mean	SD	p-value	SRM
ND parents																							
SF-6D	13	-0.04	0.07	0.14	-0.499a		9	-0.02	0.13	0.72	-0.12		13	-0.02	0.05	0.28	-0.31a		9	-0.05	0.15	0.38	-0.31a
SF-12 PCS	13	-1.64	11.96	0.63	-0.14		9	-3.49	6.89	0.17	-0.50b		13	-1.57	11.68	0.64	-0.13		9	-3.59	7.55	0.19	-0.48a
SF-12 MCS	13	-0.19	15.41	0.97	-0.01		9	1.25	12.30	0.77	0.10		13	1.34	14.84	0.75	0.09		9	-0.95	13.24	0.84	-0.07

*Note*: PROMs: patient-reported outcome measures; ND: neurodevelopmental disorders.

The values of 0.2, 0.5 and 0.8 represent the cut-off points for small (a), moderate (b), and large (c) standardised response mean (SRM) effect sizes

## Discussion

This study assessed the responsiveness of three generic PROMs (SF-6D, EQ-5D-5L, AQoL-8D) to diagnostic, personal, and clinical outcomes of genomic testing using prospectively collected data from adult patients (n = 170) and parents of affected children (n = 84) recruited within the Australian and Melbourne Genomics Health Alliances, 2016–2019. The SF-6D and EQ-5D-5L descriptive systems were not responsive to the selected anchors of diagnostic, personal, and clinical outcomes of genomic testing. While some positive changes were observed in mental health-related domains, the measures primarily captured the progressive nature of the conditions and the declining physical health of adult patients. Similar patterns were also observed among parents of children with NDs and GKDs, which may reflect additional physical needs or symptoms experienced by parents to care for their child [29, 56]. Even when controlling for baseline physical conditions, the mental health domains in SF-6D did not capture the broader non-health benefits among parents of children with NDs and GKDs and adult GKD patients (Appendix I). Of note, among CNDs participants who received a confirmed molecular diagnosis, we observed opposite trajectories for the EQ-5D-5L utility scores and EQ VAS scores, with the EQ VAS scores demonstrating improvements in subjective quality of life in light of declining objective valuation of health. The AQoL-8D, which reflects a broader definition of health encompassing aspects of wellbeing, showed improvements in the quality of life of DCM patients following a diagnosis due to improvements in the psychosocial domains of the measure. No differences, however, were identified between patients reporting personal utility from genomic testing and those who did not.

This study reports the baseline utility scores for each cohort and found that the mean utility scores were lower than general population in Australia, which was expected. Utility scores for patients or parents of affected children ranged between 0.70 and 0.72, which were between 0.05 and 0.2 lower than Australian population norms by corresponding measures [57–59]. The utility scores in our cohorts were also lower compared to other rare diseases in Australia. For example, mean utility score for children with Cystic Fibrosis in Australian measured by QWB was 0.76 [60]. A recent study in Hong Kong reported a mean utility score of pediatric and adult patients with rare diseases (13 rare diseases categories) was 0.53 [61], which was lower than our study. This may be due to differences in conditions, adults and children, PROMs and different characteristics of value sets used. In our study, we also found lower baseline and follow up utility scores among GKDs parents compared to GKD adult patients. One explanation relates to the spillover effects on carers’ health. For example, Wu et al. examined the spillover effects using data from NDs and GKDs cohort and found that the mean magnitude of HRQoL loss in parents was estimated to be 33% of the HRQoL loss observed in children [29]. Other studies also identified that parents of children with rare disease have additional physical, psychological, emotional and social need [56, 62]. Alternative explanations on the differences in utilities between GKD parents and GKD adult patients may include heterogeneity in the disease progression, severity of symptoms and adaptation to the condition. For example, a study from Switzerland found that patients with acquired kidney diseases had lower mental HRQoL than patients with congenital anomalies of the kidney, and that adults after pediatric kidney failure have lower physical, but similar mental HRQoL compared to the general population [63]. There is evidence that for many anomalies, HRQL has been shown to be worst for the youngest children and improves over time [64].

This study benefited from the use of different PROMs in different clinical contexts involving both childhood- and adult-onset genetic conditions. However, some caveats should be noted when interpreting our results. Our studies were undertaken in relatively small samples of patients or parents experiencing specific rare diseases and who responded to baseline and follow-up surveys with complete information on the PROMs and the personal utility questions. This is particularly the case for EQ-5D-5L and AQoL-8D who were applied in a single condition only. The small sample size may limit the statistical analysis and results. In addition, we used three anchors to assess the responsiveness. However, personal utility and clinical utility may be linked to diagnostic outcome. We found moderate to strong correlations between diagnostic utility and personal utility, diagnostic utility and clinical utility in NDs cohort, moderate correlations between diagnostic utility and personal utility in GKDs cohort. On the other hand, the information generated from genomic testing may also lead to multiple and different outcomes for each respondent, and variations in the length of diagnostic odyssey may also contribute to the large standard deviation in health utilities observed among participants and some different patterns across different conditions.

Another limitation is that we used different PROMs for different conditions, thus no direct comparison can be made on the responsiveness of the instruments. We used a generic PROM (i.e. SF-12v2) to measure parental HRQoL. There are broader measures of health and wellbeing (e.g., EQ-HWB and AQoL-8D) and carer measures (e.g. ASCOT-Carer) that might be more sensitive and responsive to capturing the impact of genomic testing on parental HRQoL. Furthermore, we used SF-12v2 in NDs and GKDs sample, but we used the value set developed for SF-12 for the UK general population [31]. A newer version of SF-6D (SF-6Dv2) was developed; however, SF-6Dv2 utility scores cannot be directly applied to SF-12v2 because descriptive system of SF-6Dv2 was not restricted to SF-12v2 items. There are substantial differences in the range of utilities between SF-6Dv1 (e.g. SF-6Dv1 does not have negative values whereas the minimal value for SF-6Dv2 was -0.574 in UK value set and − 0.685 in Australian value set [35, 65]. The magnitude of declines in health utilities in NDs and GKDs sample may be underestimated.

Finally, duration is limited to approximately 7 months, which was deemed sufficient to reflect the benefits of diagnostic genomic testing. Longer-term follow up would have enabled the capture of downstream improvements in health status associated with changes in clinical management. However, it might be not feasible to determine a standard longer-term follow-up point because this varies substantially between different conditions, treatment trajectories and individual adaption to the symptoms and conditions. Variations may also exist in the rate of progression, severity of symptoms, etc. The three PROMs we used in the study have different recall period: ‘today’ (EQ-5D-5L), ‘past 4 weeks’ (SF-12v2), without specifying recall period (AQoL-8D). Future research examining PROM’s recall period and its capacity to capture change in rare conditions would further improve the evidence base on the responsiveness of generic PROMs in the context of rare conditions.

Even though our findings suggest that generic PROMs and estimated utility scores may not fully quantify the non-health and process outcomes of genomic testing, our findings do not imply an immediate move away from the current QALY-led approach recommended by HTA agencies. As mentioned earlier, there are many methodological challenges in evaluating the outcomes associated with genomics [4, 14, 21, 23, 66], and thus more empirical evidence is needed. Assessments of validity and responsiveness using larger samples, potentially from multiple countries, using different conditions, including children and adults, and providing head-to-head comparisons of different measures would provide further valuable insights. Our ongoing research are going to address this by establishing new cohorts and continue to improve the evidence base. Nevertheless, HTA agencies arguably need to find ways of incorporating evidence on non-health and process benefits when informing healthcare priorities. Methods for estimating these benefits, such as discrete choice experiments and contingent valuation approaches, are well established and can provide important insights that may currently be missed. Importantly, all stakeholders need to be aware of the opportunity costs involved in the established value judgments determined by HTA bodies. Omitting the value of non-health and process outcomes to patients has important health, economic, and equity implications. In this process, health economists should work proactively with clinical researchers, service providers, policymakers and patients and their families to contribute to translational research in genomics and inform decision-making in the resource allocation process.

## Conclusion

Generic PRO measures may lack responsiveness to the diagnostic, clinical and personal outcomes of genomics, but further research is needed to establish their measurement properties and relevant evaluative space in the context of rare conditions. Expected declines in physical health of people experiencing rare conditions may further challenge the conventional application of quality of life assessments.

### Electronic supplementary material

Below is the link to the electronic supplementary material.

**Supplementary Material 1:** Clinial background, Construction of personal utility indicators, Sample characteristics and Additional analyses on changes in health outcomes by conditions

## Data Availability

The datasets generated during and analysed during the current study are not publicly available and restrictions apply to the availability of these data. Data are however available from the authors upon reasonable request and with permission of the above-mentioned Human Research Ethics Committees.

## Abbreviations

PROMs: patient reported outcome measures
PRO: patient reported outcome
HTA: Health Technology Assessment
QALYs: Quality-Adjusted Life-Years
NDs: neurodevelopmental disorders
GKDs: genetic kidney diseases
CNDs: neurodegenerative disorders
DCM: dilated cardiomyopathy
DASS: Depression, Anxiety and Stress Scale
Neuro-Qol: Neurology-Quality of Life
PAW: Positive Affect and Well-being
