# Supplementary material for: QALYs and rare diseases: exploring the responsiveness of SF-6D, EQ-5D-5L and AQoL-8D following genomic testing for childhood and adult-onset rare genetic conditions in Australia
Source: Health Qual Life Outcomes. 2023 Dec 12;21:132. doi: 10.1186/s12955-023-02216-9 (PMC10717517; doi:10.1186/s12955-023-02216-9)
Supplement: Supplementary file 1 — Supplementary Material 1: Clinial background, Construction of personal utility indicators, Sample characteristics and Additional analyses on changes in health outcomes by conditions [file 12955_2023_2216_MOESM1_ESM.docx]

Appendix A Clinical background of each cohort study

| Table A1 Clinical background of each cohort study | | | | | | |
| --- | --- | --- | --- | --- | --- | --- |
| Flagship | Mitochondrial Disorder (ND) | Epileptic Encephalopathy （ND） | Brain Malformation （ND） | Genetic Kidney Diseases (GKDs) | Complex neurological diseases (CNDs) | Dilated cardiomyopathy (DCM) |
| Duration | 2017-2019 | 2017-2019 | 2017-2019 | 2017-2019 | 2017-2018 | 2016-2017 |
| Recruitment places | NSW, QLD, SA,VIC, WA | NSW, QLD, SA,TAS, VIC, WA | NSW, QLD, SA, VIC, WA, NT | NSW, QLD, SA,VIC, WA | VIC | VIC |
| NGS | WES + mtDNA or WGS | WES | WES | WES or Panel | WES | WES |
| Inclusion criteria | a potential recruit is categorised as probable (score 5-7) or definite (score 8-12) based on the Nijmegen clinical criteria for mitochondrial disease | a)Onset of seizures <18years  b)Relatively frequent seizures (not a fixed frequency)  c) evelopmental plateau or regression  d)Epileptiform activity on EEG | a) Brain malformation diagnosed by MRI  b) Malformation of presumed genetic basis given the clinical and MRI features or family history  c)Normal chromosome microarray  d)Normal CMV PCR from neonatal Guthrie card in the case of polymicrogyria | Patients were recruited if their clinical presentation was consistent with a monogenic cause (e.g., glomerular, ubulointerstitial, or cystic renal disease) and prioritized if they met one of the following criteria: family history of renal disease, syndromic features, or childhood onset of disease. | a) age at recruitment greater than 11 years, b) symptoms and signs of one of the above clinical syndromes, c) age at onset of symptoms between 2 and 60 years (inclusive). | patients with idiopathic DCM who were either (1) diagnosed under the age of 40 years, or (2) had a family history (≥2 members per family including the proband) of DCM and/or early (<35 years) sudden unexplained death. |
| Exclusion criteria | any one or more of below：  An indication that there is another non-mitochondrial disease diagnosis from other investigatory testing  Presence of a molecular diagnosis  Patients who have not had all investigations listed in the clinical data section | a) Not static intellectual disability with epilepsy  b) No epileptiform activity on EEG | a) Brain malformation where a non-genetic cause is known or suspected based on the antenatal history (e.g. infection) or imaging appearance  b) Definitive diagnosis by non-genetic means such as biochemical studies | Patients who did not meet these criteria were considered at the discretion of the MDT. Patients with phenotypes where the likelihood of a monogenic cause is low, were only included if they had extrarenal features. Patients with a pre-existing molecularly confirmed genetic diagnosis or a phenotype and family history suggestive of typical autosomal dominant polycystic kidney disease (ADPKD) were excluded. | a) previous genetic testing in the participant or family members (except tests for conditions not reliably detectable using current ES technology, such as chromosomal microarray and repeat expansion testing, b) a specific single-gene disorder highly likely, c) where the likelihood of a monogenic cause was deemed unlikely (e.g. much more likely to be non-genetic aetiology, or functional neurological disorder) based on multidisciplinary review by the study team and liaison with the referrer and d) where the majority of clinical care was not based in Victoria, Australia | Patients with known nongenetic causes or patients who had undergone prior genetic testing involving cardiomyopathy‐related genes were excluded from the study |
| HRQoL instruments | SF-12v2 | SF-12v2 | SF-12v2 | SF-12v2 | EQ-5D-5L; DASS-21;  Neuro-Qol | AQoL-8D |
| Baseline survey (Survey 1) | after attending genetics clinics at which patients are offered and consent to testing | | | 2–3 weeks after attending the renal genetics clinics at which patients are offered and consent to testing | after attending genetics clinics at which patients are offered and consent to testing | after attending genetics clinics at which patients are offered and consent to testing |
| Follow up survey (Survey 2) | following the receipt of genomic test results. | | | following the receipt of genomic test results. | 2-3 weeks after receiving the test results | 2-3 weeks after receiving the test results |
| Reference | [[1](#_ENREF_1)] | [[1](#_ENREF_1)] | [[1](#_ENREF_1)] | [[1-4](#_ENREF_1)] | [[1](#_ENREF_1), [5](#_ENREF_5)] | [[6-8](#_ENREF_6)] |
| ND = neurodevelopmental disorders. GKDs = genetic kidney diseases; CNDs =complex neurological diseases; DCM = dilated cardiomyopathy  WES = whole-exome sequencing, WGS = whole-genome sequencing. Information and differences between WES, WGS and panel can be found here [[9-11](#_ENREF_9)]  NSW = New South Wales, VIC = Victoria, WA = Western Australia, QLD = Queensland, NT = Northern Territory, SA = South Australia, TAS = Tasmania | | | | | | |

Appendix B Construction of an indicator of personal utility and validation

All four studies collected information from patients or parents on how valuable next-generation sequencing (NGS) has been for them and their family, after the disclosure of genomic testing results. We constructed a personal utility indicator based on samples of patients who responded to relevant questions. In complex neurological diseases (CNDs) and genetic kidney diseases (GKDs) cohort study, nine statements were presented on how valuable the NGS has been for the patients and their family: (Q1) Ongoing investigations no longer necessary; (Q2) Knowing the cause/explanation for the condition; (Q3) Information for my own family planning; (Q4) Information for other members of my family; (Q5) Information for treatment/management of the condition; (Q6) Information regarding prognosis/ knowing what to expect in the future; (Q7) Have had access to the most recent advances in medicine; (Q8) I have done everything I can to improve health; and (Q9) Ability to connect with others with the same condition. In neurodevelopmental disorders (NDs) cohort study, eight questions were included in the questionnaire: Q1-Q6, Q9 and (Q10) To contribute to research. In the dilated cardiomyopathy (DCM) cohort study, 11 questions were asked: Q1-Q10 and (Q11) Data may be examined in more detail to find answer in them. Respondents were asked to answer on a 5-level scale including “extremely valuable”, “valuable”, “neutral”, “not valuable”, and “not applicable” (if the test has not provided the described impact to the question). For each question, we constructed a dummy variable for respondents who found it “extremely valuable” or “valuable”. The percentage of respondents who found value in NGS is reported in Table B1.

| Table B1 Value in genomic sequencing test among the four rare disease groups | | | | | | | | | | | |
| --- | --- | --- | --- | --- | --- | --- | --- | --- | --- | --- | --- |
|  |  | NDs parents | | GKDs adults | | GKDs parents | | CNDs | | DCM | |
|  | Value aspects | No. of respondents | % | No. of respondents | % | No. of respondents | % | No. of respondents | % | No. of respondents | % |
| Q1 | Ongoing investigations no longer necessary | 68 | 44% | 62 | 61% | 32 | 69% | 42 | 74% | 23 | 61% |
| Q2 | Knowing the cause / explanation for the condition | 68 | 68% | 75 | 84% | 34 | 85% | 63 | 73% | 26 | 81% |
| Q3 | Information for my own family planning | 61 | 67% | 57 | 77% | 26 | 62% | 48 | 69% | 23 | 65% |
| Q4 | Information for other members of my family | 65 | 63% | 80 | 85% | 36 | 81% | 70 | 86% | 32 | 91% |
| Q5 | Information for treatment / management of the condition | 68 | 53% | 73 | 82% | 36 | 86% | 58 | 76% | 29 | 72% |
| Q6 | Information regarding prognosis/ knowing what to expect in the future | 66 | 61% | 73 | 77% | 37 | 84% | 55 | 76% | 27 | 74% |
| Q7 | Have had access to the most recent advances in medicine | NA |  | 68 | 82% | 35 | 91% | 63 | 81% | 31 | 74% |
| Q8 | I have done everything I can to improve health | NA |  | 76 | 76% | 36 | 86% | 68 | 84% | 30 | 87% |
| Q9 | Ability to connect with others with the same condition | 56 | 30% | 58 | 34% | 29 | 38% | 53 | 62% | 24 | 38% |
| Q10 | To contribute to research | 80 | 85% |  |  |  |  |  |  | 34 | 97% |
| Q11 | Data be examined in more detail to find answer |  |  |  |  |  |  |  |  | 32 | 87.5% |
| Note: | The analysis was conducted among patients who responded to the value aspect questions | | | | | |  |  |  |  |  |
|  | For each value aspects questions, we did not take into account of those who answered “not applicable” | | | | | | | |  |  |  |
|  | %: percentage of respondents found NGS valuable in one aspect | | | |  |  |  |  |  |  |  |

These questions covered different aspects of value of NGS but some may be correlated. We used factor analysis to explore to what degree individual items are measuring a something in common that can have meaningful interpretations. Factor analysis produces factor loadings, which can be used as a means of item reduction (multiple items capturing the same variance or a low amount of variance can be identified and removed) and of grouping items into construct subscales or domains by their factor loadings. Factor analysis has been widely used in psychology, social science and clinical research.[[12](#_ENREF_12), [13](#_ENREF_13)] Key steps on factor analysis include: (1) Extracting initial factors. There are different ways for factor analysis, and principal factor method is one of the recommended methods. Another decision needs to be made is how many factors to include, which usually follows the Kaiser or eigenvalue criterion (eigenvalue greater than or equal to 1). (2) Factor rotation to a terminal solution, which is done to find factors that are easier to interpret. To obtain the initial solution, several restrictions are imposed, including (a) there are k common factors, (b) underlying factors are orthogonal to each other, and (c) the first factor accounts for as much variance as possible, the second factor accounts for as much of the residual variance left unexplained by the first factor, the third factor accounts for as much of the residual variance left unexplained by the first two factors, and so on [[14](#_ENREF_14)]. Before rotation, the factor loadings may not immediately be identifiable as separate factors. The object of rotation is to ensure that all variables have high loadings only on one factor. Detailed explanation and example can be found here [[15](#_ENREF_15)] Varimax (orthogonal) rotation is recommended rotation method. (3) Calculation of factor scores and use in further analysis.[[14](#_ENREF_14)] There are non-refined methods (such as sum scores by factor, sum score above a cut-off value) and refined methods (such as regression scores and Bartlett scores).[[16](#_ENREF_16)] There are strengths and considerations of different approaches to calculate factor scores.

We separately conducted factor analysis on value perspectives of NGS for each cohort using the principal factor method. In the factor analysis for parents of affected children with confirmed or suspected NDs, five items (Q 2, 3, 4, 5 and 6) had substantial loadings and we retained one factor on providing information.

|  | Table B2 Rotated factor loadings (pattern matrix) and unique variances among NDs parents | | |
| --- | --- | --- | --- |
|  | Variable | Factor1 | Uniqueness |
| Q1 | Ongoing investigations no longer necessary | 0.70 | 0.51 |
| Q2 | Knowing the cause / explanation for the condition | **0.91** | 0.17 |
| Q3 | Information for my own family planning | **0.76** | 0.42 |
| Q4 | Information for other members of my family | **0.79** | 0.38 |
| Q5 | Information for treatment / management of my child's condition | **0.87** | 0.24 |
| Q6 | Information regarding prognosis/ knowing what to expect in the future for my child's health | **0.86** | 0.26 |
| Q9 | Ability to connect with others with the same condition | 0.65 | 0.58 |
| Q10 | To contribute to research | 0.29 | 0.91 |
|  | Note: N=80. KMO coefficients: 0.90, suggesting the acceptance of factor analysis is meritorious | | |

| Table B3 Rotated factor loadings (pattern matrix) and unique variances among GKDs parents and adult patients, CNDs and DCM patients | | | | | | | | | | |
| --- | --- | --- | --- | --- | --- | --- | --- | --- | --- | --- |
|  |  | GKDs patients and parents (all=120) | | | CNDs (N=74) | | | DCM (N=30) | | |
|  | Variable | Factor1 | Factor2 | Uniqueness | Factor1 | Factor2 | Uniqueness | Factor1 | Factor2 | Uniqueness |
| Q1 | Ongoing investigations no longer necessary | 0.33 | 0.31 | 0.81 | 0.32 | 0.32 | 0.79 | 0.42 | 0.02 | 0.59 |
| Q2 | Knowing the cause / explanation for the condition | **0.80** | 0.12 | 0.37 | **0.82** | 0.3 | 0.23 | **0.85** | 0.25 | 0.08 |
| Q3 | Information for my own family planning | 0.44 | 0.26 | 0.73 | 0.63 | 0.02 | 0.6 | 0.16 | 0.04 | 0.48 |
| Q4 | Information for other members of my family | 0.64 | 0.27 | 0.52 | 0.74 | 0.25 | 0.39 | 0.65 | 0.37 | 0.24 |
| Q5 | Information for treatment / management of the condition | **0.78** | 0.26 | 0.33 | **0.84** | 0.26 | 0.23 | **0.91** | 0.24 | 0.06 |
| Q6 | Information regarding prognosis/ knowing what to expect in the future | **0.78** | 0.28 | 0.31 | **0.83** | 0.24 | 0.26 | **0.91** | 0.2 | 0.1 |
| Q7 | Have had access to the most recent advances in medicine | 0.33 | **0.58** | 0.56 | 0.34 | **0.68** | 0.42 | 0.37 | **0.78** | 0.25 |
| Q8 | I have done everything I can to improve health | 0.29 | **0.59** | 0.56 | 0.26 | **0.65** | 0.51 | 0.29 | **0.8** | 0.24 |
| Q9 | Ability to connect with others with the same condition | 0.35 | 0.49 | 0.62 | 0.46 | 0.53 | 0.51 | 0.55 | 0.25 | 0.32 |
| Q10 | To contribute to research |  |  |  |  |  |  | 0.28 | 0.07 | 0.38 |
| Q11 | Data be examined in more detail to find answer |  |  |  |  |  |  | 0.27 | 0 | 0.35 |
| Note: We conducted factor analysis on these questions using the principal factor method for each study respectively. | | | | | | | | | | |
| KMO coefficient in factor analysis among GKDs parents and adult patients = 0.85, suggesting the acceptance of factor analysis is meritorious | | | | | | | | | | |
| KMO coefficient in factor analysis among CNDs patients = 0.84, suggesting the acceptance of factor analysis is meritorious | | | | | | | | | | |
| KMO coefficient in factor analysis among DCM patients = 0.78, suggesting the acceptance of factor analysis is middling | | | | | | | | | | |

In GKDs, CNDs and DCM patients, we were able to retain two main factors with meaningful interpretations. Three items (Q2, Q5 and Q6) have substantial loadings on the first factor, representing the value in providing information on causes, treatment and prognosis; two items (Q7 and Q8) had significant loadings on the second, reflecting value in maximising chances to improve health. The rotated factor loadings for the GKDs parents and adult patients, CNDs and DCM patients are presented in Table B3. We conducted Kaiser-Meyer-Olkin (KMO) estimation, and the coefficients were between 0.78 to 0.85, suggesting the acceptance of factor analysis was middling and meritorious.

Given these factor loadings, we obtained factor scores using the regression method. We also constructed a factor-based score (i.e. sum score by factor which is an non-refined method) for each factor based on the total number of questions to which respondents answered “valuable” or “extremely valuable”. For NDs parents, one factor-based score was generated based on items 2-6, ranging from 0 to 5. For GKDs parents and adult patients, CNDs and DCM patients, the factor-based score for the first factor was generated based on items 2, 5 and 6 (score ranges from 0 to 3), and the factor-based score for the second factor based on items 7 or 8 (score ranges from 0 to 2). Based on the factor-based score, we constructed dummy variables to represent whether the respondents found value for each retained factor (=1 if the factor-based score >0).

We confirmed the validity of our constructed factor-based scores by reporting the distribution by the presence of diagnosis, as shown Tables B4-B8 for each disease respectively. The percentage of respondents who found NGS valuable was much higher among those who received a confirmed diagnosis compared to those who did not. We also examined the correlation between our constructed factor-based scores and obtained factor scores post factor analysis using the Spearman test. Among NDs parents, the correlation coefficient for the value aspect in providing information is -0.91. Among GKDs parents and adult patients, CNDs and DCM patients, for the value aspect in providing information, the correlation coefficients are -0.91, -0.91 and -0.89 respectively, and for the value aspect in chances to improve health, the correlation coefficients are -0.81, -0.85 and -0.86 respectively, suggesting a strong correlation.

| Table B4 The distribution of factor-based scores among NDs parents and by the presence of diagnosis | | | | | | |
| --- | --- | --- | --- | --- | --- | --- |
|  | Full sample | | Diagnosis | | No diagnosis | |
|  | N | % | N | % | N | % |
| Scores of value in providing information | |  |  |  |  |  |
| 0 (Not valuable) | 20 | 25% | 1 | 4% | 19 | 34% |
| 1 (Valuable in one item) | 11 | 14% | 1 | 4% | 10 | 18% |
| 2 (Valuable in two items) | 9 | 11% | 1 | 4% | 8 | 14% |
| 3 (Valuable in three items) | 9 | 11% | 6 | 25% | 3 | 5% |
| 4 (Valuable in four items) | 10 | 13% | 5 | 21% | 5 | 9% |
| 5 (Valuable in five items) | 21 | 26% | 10 | 42% | 11 | 20% |
| Total number of respondents | 80 |  | 24 |  | 56 |  |

| Table B5 The distribution of factor-based scores among GKDs adults patients and by the presence of diagnosis | | | | | | |
| --- | --- | --- | --- | --- | --- | --- |
|  | Full sample | | Diagnosis | | No diagnosis | |
|  | N | % | N | % | N | % |
| Scores of value in providing information | |  |  |  |  |  |
| 0 (Not valuable) | 15 | 18% | 1 | 2% | 14 | 37% |
| 1 (Valuable in one item) | 11 | 13% | 8 | 18% | 3 | 8% |
| 2 (Valuable in two items) | 10 | 12% | 6 | 14% | 5 | 11% |
| 3 (Valuable in three items) | 46 | 56% | 29 | 66% | 17 | 45% |
| Scores of value in maxmising chances to improve health | | |  |  |  |  |
| 0 (Not valuable) | 16 | 19% | 7 | 16% | 9 | 21% |
| 1 (Valuable in one item) | 22 | 27% | 12 | 27% | 10 | 26% |
| 2 (Valuable in two items) | 44 | 54% | 25 | 57% | 19 | 53% |
| Total number of respondents | 82 |  | 44 |  | 38 |  |

| Table B6 The distribution of factor-based scores among GKDs parents patients and by the presence of diagnosis | | | | | | |
| --- | --- | --- | --- | --- | --- | --- |
|  | Full sample | | Diagnosis | | No diagnosis | |
|  | N | % | N | % | N | % |
| Scores of value in providing information | |  |  |  |  |  |
| 0 (Not valuable) | 3 | 8% | 2 | 9% | 1 | 7% |
| 1 (Valuable in one item) | 5 | 13% | 3 | 13% | 2 | 13% |
| 2 (Valuable in two items) | 4 | 11% | 2 | 9% | 2 | 13% |
| 3 (Valuable in three items) | 26 | 68% | 16 | 69% | 10 | 67% |
| Scores of value in maxmising chances to improve health | | |  |  |  |  |
| 0 (Not valuable) | 2 | 5% | 2 | 9% | 0 |  |
| 1 (Valuable in one item) | 9 | 24% | 8 | 35% | 1 | 793% |
| 2 (Valuable in two items) | 27 | 71% | 13 | 56% | 15 | 94% |
| Total number of respondents | 38 |  | 23 |  | 15 |  |

| Table B7 The distribution of factor-based scores among adult CND patients and by the presence of diagnosis | | | | | | |
| --- | --- | --- | --- | --- | --- | --- |
|  | Full sample | | Diagnosis | | No diagnosis | |
|  | N | % | N | % | N | % |
| Scores of value in providing information | |  |  |  |  |  |
| 0 (Not valuable) | 25 | 34% | 1 | 6% | 24 | 43% |
| 1 (Valuable in one item) | 11 | 15% | 4 | 22% | 7 | 13% |
| 2 (Valuable in two items) | 6 | 8% | 1 | 6% | 5 | 9% |
| 3 (Valuable in three items) | 32 | 43% | 12 | 67% | 20 | 36% |
| Scores of value in maxmising chances to improve health | | |  |  |  |  |
| 0 (Not valuable) | 17 | 23% | 4 | 22% | 13 | 23% |
| 1 (Valuable in one item) | 15 | 20% | 3 | 16% | 12 | 21% |
| 2 (Valuable in two items) | 42 | 57% | 11 | 62% | 31 | 55% |
| Total number of respondents | 74 |  | 18 |  | 56 |  |

| Table B8 The distribution of factor-based scores among DCM patients and by the presence of diagnosis | | | | | | |
| --- | --- | --- | --- | --- | --- | --- |
|  | Full sample | | Diagnosis | | No diagnosis | |
|  | N | % | N | % | N | % |
| Scores of value in providing information | |  |  |  |  |  |
| 0 (Not valuable) | 12 | 40% | 1 | 14% | 11 | 48% |
| 1 (Valuable in one item) | 1 | 3% | 1 | 14% | 0 | 0% |
| 2 (Valuable in two items) | 2 | 7% | 1 | 14% | 1 | 4% |
| 3 (Valuable in three items) | 15 | 50% | 4 | 57% | 11 | 48% |
| Scores of value in maxmising chances to improve health | | |  |  |  |  |
| 0 (Not valuable) | 7 | 23% | 2 | 29% | 5 | 22% |
| 1 (Valuable in one item) | 5 | 17% | 2 | 29% | 3 | 13% |
| 2 (Valuable in two items) | 18 | 60% | 3 | 42% | 15 | 65% |
| Total number of respondents | 30 |  | 7 |  | 23 |  |

Appendix C Sample characteristics


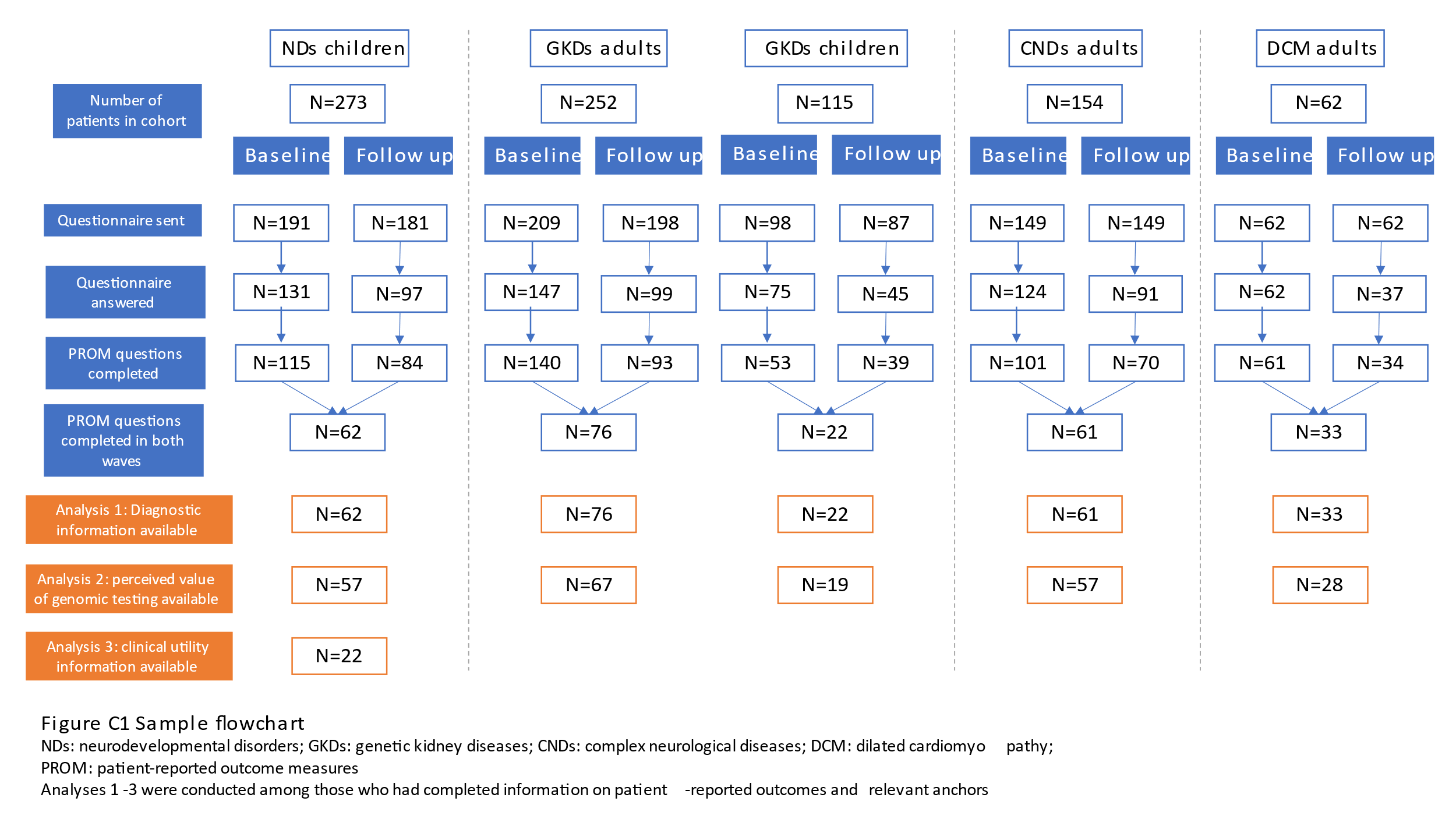


Figure C1 provides a sample flowchart for each cohort. Analyses 1 -3 were conducted among those who had completed information on patient-reported outcomes and relevant anchors. As demonstrated in the sample flowchart, not all the patients in the cohort received questionnaires, particularly in NDs and GKDs. Some common reasons for not receiving the surveys were (1) testing result disclosed before the baseline survey could be sent, (2) result not disclosed to the patients, (3) withdrawn, (4) deceased, (5) no email address, and (6) difficulty with English. We did not force answers to the questionnaires, therefore not everyone who responded to the survey completed questions on PROs. Potential reasons for not completing PROMs may include: difficulties and burden in responses, not feasible/relevant to their condition, mode of administration [[17](#_ENREF_17), [18](#_ENREF_18)], which may indicate the challenges in using traditional PROMs in genomic testing.

| Table C1 Sample characteristics at baseline among respondents who completed generic patient-reported outcome measures questionnaire at baseline and follow-up | | | | | | | | | | |
| --- | --- | --- | --- | --- | --- | --- | --- | --- | --- | --- |
|  | NDs parents (N=62) | | GKDs adults (N=76) | | GKDs parents (N=22) | | CNDs adults (N=61) | | DCM adults (N=33) | |
| Variables | Frequency | % | Frequency | % | Frequency | % | Frequency | % | Frequency | % |
| Age |  |  |  |  |  |  |  |  |  |  |
| < 30 | 7 | 11% | 17 | 22% | 0 | 0% | 2 | 3% | 0 | 0% |
| 30-44 | 48 | 77% | 22 | 29% | 11 | 50% | 12 | 20% | 8 | 24% |
| 45-64 | 7 | 11% | 29 | 38% | 10 | 45% | 32 | 52% | 17 | 52% |
| >=65 | 0 | 0% | 8 | 11% | 1 | 5% | 15 | 25% | 8 | 24% |
| Gender |  |  |  |  |  |  |  |  |  |  |
| Male | 8 | 13% | 31 | 41% | 20 | 95% | 26 | 43% | 12 | 36% |
| Female | 54 | 87% | 45 | 59% | 1 | 5% | 35 | 57% | 21 | 64% |
| Education |  |  |  |  |  |  |  |  |  |  |
| Year 11 or below | 4 | 6% | 9 | 12% | 2 | 9% | 14 | 23% | 10 | 30% |
| Year 12 or equivalent | 16 | 26% | 16 | 21% | 2 | 9% | 10 | 16% | 5 | 15% |
| Certificate/Diploma | 12 | 19% | 20 | 26% | 3 | 14% | 17 | 28% | 9 | 27% |
| Bachelor | 16 | 26% | 23 | 30% | 9 | 41% | 9 | 15% | 2 | 6% |
| Graduate and above | 14 | 23% | 8 | 11% | 6 | 27% | 11 | 18% | 6 | 18% |
| Diagnostic outcome - final diagnosis | | | | | | | | | | |
| Diagnosis | 20 | 32% | 40 | 53% | 11 | 50% | 10 | 16% | 6 | 18% |
| No diagnosis | 42 | 68% | 36 | 47% | 11 | 50% | 51 | 84% | 27 | 82% |
| Quality of life* | Mean | SD | Mean | SD | Mean | SD | Mean | SD | Mean | SD |
| Baseline | 0.72 | 0.12 | 0.72 | 0.13 | 0.70 | 0.12 | 0.71 | 0.29 | 0.71 | 0.18 |
| Follow-up | 0.69 | 0.13 | 0.71 | 0.14 | 0.69 | 0.11 | 0.70 | 0.28 | 0.72 | 0.17 |
| Note: NDs: neurodevelopmental disorders; GKDs: genetic kidney diseases; CNDs: complex neurological diseases; DCM: dilated cardiomyopathy  The following HRQoL measures were used for each cohort and we reported utility scores (on a 0-1 scale) calculated from each: SF-6D(Vsf-12) were used for NDs and GKDs. EQ-5D-5L was used for CNDs. AQoL-8D was used for DCM | | | | | | | | | | |

In our analytical sample, mean age of parents of children affected by NDs and GKDs were 37.2 and 43.1 years respectively, and more than 85% of the respondents were female. The diagnostic rates among both cohort were 32% and 50% which were fairly close to those in the recruited sample as shown in Table C2. Mean age of adult patients with GKDs, CNDs and DCM were 43.3, 53.4 and 53.5 years respectively. The diagnostic rates were 53%, 16% and 18% among adults patients with GKD, CND and DCM in our analytical sample respectively, while the numbers among the recruited patients were 45%, 22% and 13%.

Table C1 also presents the baseline utility scores for each cohort and the mean utility scores were lower than general population in Australia. Utility scores for parents of children affected by NDs and GKDs were 0.72 (SD 0.12) and 0.70 (SD 0.12) measured by SF-6D respectively, which was lower than the utility scores for parents of Australian children aged 11-12 years (0.81 SD 0.16) measured by AQol-8D.[[19](#_ENREF_19)] Mean SF-6D utility for GKD adult patients in our sample was 0.72 (SD 0.13), whereas the average utility score for an Australian aged 41-50 years old was 0.77 measured by SF-6D.[[20](#_ENREF_20)] Utility score for CNDs adult patients at baseline in our study was 0.71 (SD 0.29) measured using EQ-5D-5L in our sample. For Australian general population the mean EQ-5D-5L utility score was 0.91 and that for an Australian ages between 45-54 years was 0.89 (which is more close to the mean age of adult patients with CNDs in our sample).[[21](#_ENREF_21)] Utility score for adult patients with DCM was 0.71 (SD 0.18) measured by AQoL-8D, which was lower than the population norm for an average Australian ages between 45-54 years old (0.769).[[22](#_ENREF_22)]

| Table C2 Sample characteristics at baseline among recruited participants who have been informed of the test outcome | | | | | | | | | | |
| --- | --- | --- | --- | --- | --- | --- | --- | --- | --- | --- |
|  | NDs parents (N=273) | | GKDs adults (N=252) | | GKDs parents (N=115) | | CNDs adults (N=154) | | DCM adults (N=62) | |
| Variables | Frequency | % | Frequency | % | Frequency | % | Frequency | % | Frequency | % |
| Age | (N=273) |  | (N=142) |  | (N=73) |  | (N=154) |  | (N=62) |  |
| < 30 | 18 | 7% | 29 | 20% | 3 | 4% | 5 | 3% | 2 | 3% |
| 30-44 | 90 | 33% | 44 | 31% | 45 | 62% | 27 | 18% | 17 | 27% |
| 45-64 | 17 | 6% | 56 | 39% | 24 | 33% | 66 | 43% | 32 | 52% |
| >=65 | 148 | 54% | 13 | 9% | 1 | 1% | 56 | 36% | 11 | 18% |
| Gender | (N=127) |  | (N=147) |  | (N=71) |  | (N=125) |  | (N=61) |  |
| Male | 16 | 13% | 62 | 42% | 8 | 11% | 57 | 46% | 33 | 54% |
| Female | 111 | 87% | 83 | 56% | 63 | 89% | 68 | 54% | 28 | 46% |
| Other |  |  | 2 | 1% |  |  |  |  |  |  |
| Education | (N=126) |  | (N=146) |  | (N=74) |  | (N=123) |  | (N=61) |  |
| Year 11 or below | 16 | 13% | 25 | 17% | 7 | 9% | 25 | 20% | 18 | 30% |
| Year 12 or equivalent | 24 | 19% | 29 | 20% | 6 | 8% | 32 | 26% | 15 | 25% |
| Certificate/Diploma | 39 | 31% | 40 | 27% | 15 | 20% | 31 | 25% | 13 | 21% |
| Bachelor | 25 | 20% | 32 | 22% | 24 | 32% | 16 | 13% | 5 | 8% |
| Graduate and above | 22 | 17% | 20 | 14% | 20 | 27% | 18 | 15% | 7 | 11% |
| Other |  |  |  |  | 1 | 1% | 1 | 1% | 3 | 5% |
| Diagnostic outcome - final diagnosis | | | | | | | | | | |
|  | (N=273) |  | (N=252) |  | (N=115) |  | (N=154) |  | (N=62) |  |
| Diagnosis | 92 | 34% | 114 | 45% | 57 | 50% | 34 | 22% | 8 | 13% |
| No diagnosis | 181 | 66% | 138 | 55% | 58 | 50% | 120 | 78% | 54 | 87% |
| Note: NDs: neurodevelopmental disorders; GKDs: genetic kidney diseases; CNDs: complex neurological diseases; DCM: dilated cardiomyopathy  The following HRQoL measures were used for each cohort and we reported utility scores (on a 0-1 scale) calculated from each: SF-6D(Vsf-12) were used for NDs and GKDs. EQ-5D-5L was used for CNDs. AQoL-8D was used for DCM | | | | | | | | | | |

Appendix D Additional analyses on changes in health outcomes before and after genomic sequencing among neurodevelopment disorders (NDs) parents

We assessed the overall change in health state based on the change in SF-6D dimension scores. The SF-6D dimension scores are derived from SF-12, which contains 12 questions [[23](#_ENREF_23)]. The number of response levels of each question varies between dimensions, ranging from 4 to 6. Level “1” representing the best health states in that dimension, and the higher levels representing worse health state. The Physical Functioning and Pain dimensions have six levels; the Social Functioning, Mental Health and Vitality dimensions have five levels; and the Role Limitation dimension has only four levels. Scores in Physical Functioning, Pain and Social Functioning are used to calculate PCS scores, and Vitality, Role Limitation and Mental Health are used to produce MCS scores.

To assess the impact of on health profiles, we summarise the change in SF-12 profile data using the Paretian Classification of Health Change (PCHC) approach. PCHC is based on the principles of a Pareto improvement in Welfare Economics. It has been first developed and used in analysing EQ-5D profile data. We adopt the approach and use in SF-12. A health state is considered to be ‘better’ than another if it is better on at least one dimension and is no worse on any other dimension. A health state is considered to be ‘worse’ than another if it is worse in at least one dimension and is no better in any other dimension. The “mixed” changes in health refer to that better in at least one dimension, but worse in at least one other [[24](#_ENREF_24)]. PCHC shows the changes in health states without introducing any preference weights [[24](#_ENREF_24)].

Table D1 shows that all parents of children affected by NDs reported problems in at least one dimension. A higher proportion of parents experienced a worsening health state (40%) compared to those had an improvement in health state (26%).

| Table D1 Changes in health state according to the PCHC, taking account of those with no problems | | | | | | |
| --- | --- | --- | --- | --- | --- | --- |
|  | Full sample | | Diagnosis | | No diagnosis | |
|  | N | % | N | % | N | % |
| Best possible health states | 0 | 0% | 0 | 0% | 0 | 0% |
| Total with problems | 62 | 100% | 20 | 100% | 42 | 100% |
| No change | 3 | 5% | 0 | 0% | 3 | 7% |
| Improve | 16 | 26% | 5 | 25% | 11 | 26% |
| Worsen | 25 | 40% | 4 | 20% | 21 | 50% |
| Mixed change | 18 | 29% | 11 | 55% | 7 | 17% |

In line with the findings that the size of decrease in health utility was smaller among parents of children with a molecular diagnosis, we also found that the percentage of parents with worsen health states was much lower compared to no diagnosis group (20% vs 50%). We further explored the patterns in changes in health states. As shown in Table D2, among the full sample of 62 parents, 21 parents improved in health utility. Of these, 20 parents (95%) improved on at least one mental health related domain while one improved only on physical health domain (bodily pain). Four parents did not report change in health states. Among the remaining 37 parents who had declines in health utility (Table D3), 25 had worsen health state on at least one dimensions and 12 had mixed changes on health states (5 of the 12 had improvements on mental health component).

| Table D2 SF-6D dimension levels before and after genomic testing among NDs parents who had increased SF-6D utilities | | | | | | |
| --- | --- | --- | --- | --- | --- | --- |
| Phenotype | Diagnostic Outcomes | Baseline | Follow up | Change in health utility | Change in health state according to the PCHC | |
| Mito | Unconfirmed | 132322 | 133233 | 0.039 | Mixed | Pain |
| Mito | Unconfirmed | 112123 | 111123 | 0.063 | Improve | Social |
| Mito | Unconfirmed | 132122 | 111122 | 0.203 | Improve | Role Social |
| Mito | Confirmed | 133133 | 132122 | 0.003 | Improve | Social Mental Vitality |
| Mito | Confirmed | 145355 | 343434 | 0.05 | Mixed | Social Mental Vitality |
| Mito | Confirmed | 132333 | 131122 | 0.105 | Improve | Social Pain Mental Vitality |
| EE | Unconfirmed | 143134 | 132123 | 0.003 | Improve | Role Social Mental Vitality |
| EE | Unconfirmed | 134145 | 142333 | 0.058 | Mixed | Social Mental Vitality |
| EE | Unconfirmed | 131114 | 121112 | 0.077 | Improve | Role Vitality |
| EE | Unconfirmed | 133134 | 111123 | 0.206 | Improve | Role Social Mental Vitality |
| EE | Unconfirmed | 143133 | 111112 | 0.265 | Improve | Role Social Mental Vitality |
| EE | Unconfirmed | 143133 | 111112 | 0.265 | Improve | Role Social Mental Vitality |
| EE | Confirmed | 245335 | 144333 | 0.04 | Improve | Physical Social Vitality |
| EE | Confirmed | 131124 | 112122 | 0.077 | Mixed | Role Vitality |
| BM | Unconfirmed | 133133 | 132122 | 0.003 | Improve | Social Mental Vitality |
| BM | Unconfirmed | 143235 | 112124 | 0.094 | Improve | Role Social Pain Mental Vitality |
| BM | Unconfirmed | 143333 | 111132 | 0.248 | Improve | Role Social Pain Vitality |
| BM | Confirmed | 143234 | 142123 | 0.003 | Improve | Social Pain Mental Vitality |
| BM | Confirmed | 143243 | 143233 | 0.054 | Improve | Mental |
| BM | Confirmed | 131132 | 121123 | 0.077 | Mixed | Role Mental |
| BM | Confirmed | 121323 | 112113 | 0.101 | Mixed | Role Pain Mental |
| EE: epileptic encephalopathy; BM brain malformations | | | | | | |

| Table D3 SF-6D dimension levels before and after genomic testing among NDs parents who had declined SF-6D utilities | | | | | | |
| --- | --- | --- | --- | --- | --- | --- |
| Phenotype | Diagnostic Outcomes | Baseline | Follow up | Change in health utility | Change in health state according to the PCHC | |
| Mito | Unconfirmed | 242223 | 344524 | -0.2 | Worsen |  |
| Mito | Unconfirmed | 142224 | 244243 | -0.072 | Mixed | Vitality |
| Mito | Unconfirmed | 142223 | 242323 | -0.042 | Worsen |  |
| Mito | Confirmed | 243234 | 142244 | -0.051 | Mixed | Physical Social |
| Mito | Confirmed | 243534 | 245445 | -0.049 | Mixed | Pain |
| Mito | Confirmed | 242433 | 243423 | -0.003 | Mixed | Mental |
| EE | Unconfirmed | 111112 | 133122 | -0.265 | Worsen |  |
| EE | Unconfirmed | 221212 | 232222 | -0.199 | Worsen |  |
| EE | Unconfirmed | 121112 | 142132 | -0.199 | Worsen |  |
| EE | Unconfirmed | 132134 | 244454 | -0.17 | Worsen |  |
| EE | Unconfirmed | 133333 | 243543 | -0.149 | Worsen |  |
| EE | Unconfirmed | 112122 | 132122 | -0.14 | Worsen |  |
| EE | Unconfirmed | 121222 | 142223 | -0.14 | Worsen |  |
| EE | Unconfirmed | 111122 | 131122 | -0.14 | Worsen |  |
| EE | Unconfirmed | 111122 | 131133 | -0.14 | Worsen |  |
| EE | Unconfirmed | 133233 | 243433 | -0.077 | Worsen |  |
| EE | Unconfirmed | 131123 | 132133 | -0.063 | Worsen |  |
| EE | Unconfirmed | 244335 | 244433 | -0.007 | Mixed | Vitality |
| EE | Confirmed | 111322 | 131132 | -0.098 | Mixed | Pain |
| EE | Confirmed | 121111 | 111212 | -0.015 | Mixed | Role |
| EE | Confirmed | 132122 | 143123 | -0.003 | Worsen |  |
| BM | Unconfirmed | 111322 | 133334 | -0.206 | Worsen |  |
| BM | Unconfirmed | 112213 | 243123 | -0.202 | Mixed | Pain |
| BM | Unconfirmed | 131123 | 242524 | -0.2 | Worsen |  |
| BM | Unconfirmed | 111123 | 112134 | -0.14 | Worsen |  |
| BM | Unconfirmed | 231323 | 144244 | -0.093 | Mixed | Physical Pain |
| BM | Unconfirmed | 113224 | 244234 | -0.078 | Worsen |  |
| BM | Unconfirmed | 242233 | 341344 | -0.0779999 | Mixed | Social |
| BM | Unconfirmed | 131123 | 243224 | -0.066 | Worsen |  |
| BM | Unconfirmed | 111112 | 121112 | -0.063 | Worsen |  |
| BM | Unconfirmed | 111112 | 111123 | -0.059 | Worsen |  |
| BM | Unconfirmed | 143435 | 244435 | -0.015 | Worsen |  |
| BM | Confirmed | 113131 | 144232 | -0.233 | Worsen |  |
| BM | Confirmed | 131122 | 132142 | -0.117 | Worsen |  |
| BM | Confirmed | 111122 | 112123 | -0.063 | Worsen |  |
| BM | Confirmed | 243424 | 132533 | -0.057 | Mixed | Physical Role Social Vitality |
| BM | Confirmed | 243234 | 243333 | -0.042 | Mixed | Vitality |
| EE: epileptic encephalopathy; BM brain malformations | | | | | | |

Appendix E Additional analyses on changes in health outcomes before and after genomic sequencing among genetic kidney diseases (GKDs) adult patients and parents

Table E1 and E2 show that almost all the GKD adult patients and parents reported some problems in at least one dimensions. Overall, the number of adult patients who had improved and worsening health states was very similar. However, a higher proportion of adult patients and parents of affected children with a molecular diagnosis experienced a worsening health state compared to those did not receive a diagnosis, which is consistent with our finding on changes on health utility.

| Table E1 Changes in health state according to the PCHC among adult GKDs patients, taking account of those with no problems | | | | | | |
| --- | --- | --- | --- | --- | --- | --- |
|  | Full sample | | Diagnosis | | No diagnosis | |
|  | N | % | N | % | N | % |
| Best possible health states | 0 | 0% | 0 | 0% | 0 | 0% |
| Total with problems | 76 | 100% | 40 | 100% | 36 | 100% |
| No change | 9 | 12% | 6 | 15% | 3 | 8% |
| Improve | 17 | 22% | 8 | 20% | 9 | 25% |
| Worsen | 16 | 21% | 10 | 27% | 6 | 17% |
| Mixed change | 34 | 45% | 16 | 39% | 18 | 50% |

| Table E2 Changes in health state according to the PCHC among parents of children with suspected GKDs, taking account of those with no problems | | | | | | |
| --- | --- | --- | --- | --- | --- | --- |
|  | Full sample | | Diagnosis | | No diagnosis | |
|  | N | % | N | % | N | % |
| Best possible health states | 0 | 0% | 0 | 0% | 0 | 0% |
| Total with problems | 22 | 100% | 11 | 100% | 11 | 100% |
| No change | 1 | 5% | 0 | 0% | 1 | 9% |
| Improve | 4 | 18% | 1 | 9% | 3 | 27% |
| Worsen | 8 | 36% | 5 | 45% | 3 | 27% |
| Mixed change | 9 | 41% | 5 | 45% | 4 | 36% |

Table E3 shows the SF-12 profiles and the change in health states of the GKD adult patients or parents who had increased SF-6D health utility, among which, 36 patients or parents had an increase in health utility, 22 remained unchanged and 40 had a declined health utility. Among the 36 patients or parents who had increased health utilities (Table E3), 33 improved on at least one mental health related domain while three improved only on physical health domain. Among the 40 patients or parents who had declines in health utility (Table E4), 17 improved on at least one mental health related domain.

| Table E3 SF-6D dimension levels before and after genomic testing among GKDs parents or patients who had increased SF-6D utilities | | | | | | | |
| --- | --- | --- | --- | --- | --- | --- | --- |
| Cohort | Phenotype | Diagnostic Outcomes | Baseline | Follow up | Change in health utility | Change in health state according to the PCHC | |
| Adult | Alport | Unconfirmed | 112123 | 111122 | 0.063 | Improve | Social Vitality |
| Adult | Alport | Confirmed | 244123 | 143223 | 0.015 | Mixed | Physical Social |
| Adult | Alport | Confirmed | 111123 | 111113 | 0.059 | Improve | Mental |
| Adult | Alport | Confirmed | 142225 | 242224 | 0.028 | Mixed | Vitality |
| Adult | Alport | Confirmed | 244325 | 143224 | 0.085 | Improve | Physical Social Pain Vitality |
| Adult | Alport | Confirmed | 142223 | 111313 | 0.22 | Mixed | Role Social Mental |
| Adult | Alport | Confirmed | 133133 | 132133 | 0.003 | Improve | Social |
| Adult | Complement disorder | Unconfirmed | 345535 | 345444 | 0.034 | Mixed | Pain Vitality |
| Adult | Complement disorder | Unconfirmed | 244444 | 144154 | 0.056 | Mixed | Physical Pain |
| Adult | Cystic - Cystic | Unconfirmed | 131124 | 111213 | 0.199 | Mixed | Role Mental Vitality |
| Adult | Cystic - Cystic | Unconfirmed | 132525 | 343433 | 0.04 | Mixed | Pain Vitality |
| Adult | Cystic - Cystic | Unconfirmed | 344425 | 343435 | 0.015 | Mixed | Social |
| Adult | Cystic - Cystic | Unconfirmed | 143423 | 144334 | 0.02 | Mixed | Pain |
| Adult | Cystic - Cystic | Confirmed | 231212 | 211213 | 0.14 | Mixed | Role |
| Adult | Cystic - Cystic | Confirmed | 131134 | 221133 | 0.077 | Mixed | Role Vitality |
| Adult | Cystic - Cystic | Confirmed | 122123 | 221312 | 0.08 | Mixed | Social Mental Vitality |
| Adult | Cystic - ADTKD | Unconfirmed | 111121 | 111111 | 0.059 | Improve | Mental |
| Adult | Cystic - ADTKD | Unconfirmed | 141122 | 131113 | 0.059 | Mixed | Role Mental |
| Adult | Cystic - ciliopathy | Unconfirmed | 124125 | 143134 | 0.043 | Mixed | Social Vitality |
| Adult | Glomerular disease (other) - Glomerulopathy | Confirmed | 143233 | 141133 | 0.066 | Improve | Social Pain |
| Adult | Nephrotic | Unconfirmed | 341335 | 242235 | 0.024 | Mixed | Physical Pain |
| Adult | Nephrotic | Unconfirmed | 133232 | 111132 | 0.206 | Improve | Role Social Pain |
| Adult | Nephrotic | Unconfirmed | 133134 | 113133 | 0.14 | Improve | Role Vitality |
| Adult | Tubular diseases Tubulopathy | Unconfirmed | 132222 | 131122 | 0.063 | Improve | Social Pain |
| Adult | Tubular diseases Tubulopathy | Confirmed | 111112 | 111111 | 0.078 | Improve | Vitality |
| Adult | Other | Confirmed | 121113 | 111113 | 0.063 | Improve | Role |
| Adult | Unknown | Unconfirmed | 132123 | 131133 | 0.063 | Mixed | Social |
| Adult | Unknown | Unconfirmed | 131113 | 111113 | 0.14 | Improve | Role |
| Adult | Unknown | Unconfirmed | 242334 | 133133 | 0.039 | Mixed | Physical Role Pain Vitality |
| Paediatric | Alport | Confirmed | 144154 | 145315 | 0.052 | Mixed | Mental |
| Paediatric | CAKUT - syndromic | Confirmed | 142333 | 142223 | 0.042 | Improve | Pain Mental |
| Paediatric | Cystic - Cystic | Unconfirmed | 141223 | 111111 | 0.277 | Improve | Role Pain Mental Vitality |
| Paediatric | Cystic - ciliopathy | Unconfirmed | 134232 | 133132 | 0.015 | Improve | Social Pain |
| Paediatric | Glomerular disease (other) - Glomerulopathy | Unconfirmed | 244155 | 144235 | 0.075 | Mixed | Physical Mental |
| Paediatric | Tubular diseases Tubulopathy | Unconfirmed | 344435 | 343224 | 0.12 | Improve | Social Pain Mental Vitality |
| Paediatric | Tubular diseases Tubulopathy | Confirmed | 334125 | 242334 | 0.049 | Mixed | Physical Social Vitality |

| Table E4 SF-6D dimension levels before and after genomic testing among GKDs parents or patients who had declined SF-6D utilities | | | | | | | |
| --- | --- | --- | --- | --- | --- | --- | --- |
| Cohort | Phenotype | Diagnostic Outcomes | Baseline | Follow up | Change in health utility | Change in health state according to the PCHC | |
| Adult | Alport | Unconfirmed | 235354 | 245445 | -0.042 | Mixed | Mental |
| Adult | Alport | Confirmed | 221323 | 222215 | -0.067 | Mixed | Pain Mental |
| Adult | Alport | Confirmed | 112412 | 242135 | -0.073 | Mixed | Pain |
| Adult | Alport | Confirmed | 111133 | 131124 | -0.14 | Mixed | Mental |
| Adult | Complement disorder | Unconfirmed | 142233 | 143244 | -0.057 | Worsen |  |
| Adult | Cystic - Cystic | Unconfirmed | 242233 | 243233 | -0.003 | Worsen |  |
| Adult | Cystic - Cystic | Unconfirmed | 131112 | 131132 | -0.059 | Worsen |  |
| Adult | Cystic - Cystic | Unconfirmed | 245215 | 145254 | -0.106 | Mixed | Physical Vitality |
| Adult | Cystic - Cystic | Confirmed | 131112 | 131122 | -0.059 | Worsen |  |
| Adult | Cystic - Cystic | Confirmed | 242234 | 243424 | -0.08 | Mixed | Mental |
| Adult | Cystic - Cystic | Confirmed | 131235 | 133335 | -0.108 | Worsen |  |
| Adult | Cystic - Cystic | Confirmed | 111112 | 112143 | -0.253 | Worsen |  |
| Adult | Cystic - ADTKD | Unconfirmed | 131133 | 133133 | -0.066 | Worsen |  |
| Adult | Cystic - ADTKD | Unconfirmed | 111213 | 131122 | -0.199 | Mixed | Pain Vitality |
| Adult | Cystic - ADTKD | Confirmed | 111213 | 121114 | -0.14 | Mixed | Pain |
| Adult | Cystic - ciliopathy | Unconfirmed | 121324 | 143333 | -0.066 | Mixed | Vitality |
| Adult | Glomerular disease (other) - Glomerulopathy | Confirmed | 111113 | 121124 | -0.199 | Worsen |  |
| Adult | Glomerular disease (other) - Glomerulopathy | Confirmed | 113143 | 343343 | -0.15 | Worsen |  |
| Adult | Glomerular disease (other) - Glomerulopathy | Confirmed | 321414 | 221453 | -0.089 | Mixed | Physical Vitality |
| Adult | Nephrotic | Unconfirmed | 122223 | 242423 | -0.154 | Worsen |  |
| Adult | Tubular diseases Tubulopathy | Confirmed | 132234 | 244233 | -0.018 | Mixed | Vitality |
| Adult | Tubular diseases Tubulopathy | Confirmed | 133133 | 143343 | -0.096 | Worsen |  |
| Adult | Tubular diseases Tubulopathy | Confirmed | 111112 | 123223 | -0.188 | Worsen |  |
| Adult | Tubular diseases Tubulopathy | Confirmed | 111112 | 121112 | -0.063 | Worsen |  |
| Adult | Other | Unconfirmed | 143234 | 222143 | -0.051 | Mixed | Role Social Pain Vitality |
| Adult | Other | Confirmed | 243132 | 142243 | -0.051 | Mixed | Physical Social |
| Adult | Unknown | Unconfirmed | 342424 | 323514 | -0.004 | Mixed | Role Mental |
| Paediatric | CAKUT - syndromic | Unconfirmed | 241313 | 245113 | -0.051 | Mixed | Pain |
| Paediatric | CAKUT - syndromic | Unconfirmed | 143124 | 242334 | -0.039 | Mixed | Social |
| Paediatric | Cystic - Cystic | Confirmed | 134123 | 134144 | -0.054 | Worsen |  |
| Paediatric | Cystic - Cystic | Confirmed | 132123 | 133134 | -0.003 | Worsen |  |
| Paediatric | Cystic - Cystic | Confirmed | 111122 | 111151 | -0.074 | Mixed | Vitality |
| Paediatric | Cystic - Cystic | Confirmed | 143134 | 133145 | -0.082 | Mixed | Role |
| Paediatric | Cystic - Cystic | Confirmed | 111112 | 131112 | -0.14 | Worsen |  |
| Paediatric | Cystic - Cystic | Confirmed | 141213 | 142222 | -0.122 | Mixed | Vitality |
| Paediatric | Glomerular disease (other) - Glomerulopathy | Unconfirmed | 133232 | 133333 | -0.042 | Worsen |  |
| Paediatric | Nephrotic | Unconfirmed | 111112 | 112112 | -0.063 | Worsen |  |
| Paediatric | Nephrotic | Unconfirmed | 142112 | 132132 | -0.059 | Mixed | Role |
| Paediatric | Tubular diseases Tubulopathy | Unconfirmed | 142224 | 243335 | -0.073 | Worsen |  |
| Paediatric | Tubular diseases Tubulopathy | Confirmed | 122132 | 142132 | -0.077 | Worsen |  |

Appendix F Additional analyses on changes of health outcomes before and after genomic sequencing among complex neurological diseases (CNDs) patients

To assess the impact of the choice of the value set on utility scores, we performed sensitivity analyses by summarising the change in EQ-5D profile data using the Paretian Classification of Health Change (PCHC) approach and by using a US value set.

PCHC is based on the principles of a Pareto improvement in Welfare Economics. An EQ-5D health state is considered to be ‘better’ than another if it is better on at least one dimension and is no worse on any other dimension. An EQ-5D health state is considered to be ‘worse’ than another if it is worse in at least one dimension and is no better in any other dimension. The “mixed” changes in health refer to that better in at least one dimension, but worse in at least one other [[24](#_ENREF_24)]. PCHC shows the changes in health states without introducing any preference weights [[24](#_ENREF_24)].

| Table F1 Changes in health state according to the PCHC, taking account of those with no problems | | | | | | |
| --- | --- | --- | --- | --- | --- | --- |
|  | Full sample | | Diagnosis | | No diagnosis | |
|  | N | % | N | % | N | % |
| No problems | 0 | 0% | 0 | 0% | 0 | 0% |
| Total with problems | 61 | 100% | 11 | 100% | 51 | 100% |
| No change | 9 | 15% | 3 | 30% | 6 | 12% |
| Improve | 15 | 25% | 2 | 20% | 13 | 25% |
| Worsen | 19 | 31% | 2 | 20% | 17 | 33% |
| Mixed change | 18 | 30% | 3 | 30% | 15 | 29% |

Table F1 reports the changes in health state according to the PCHC, taking account of those with no problems. All respondents reported problems in at least one dimension. A slightly higher proportion of patients reported experiencing a worsening health state (31%) compared to those who reported an improving health state (25%). This is consistent with our finding on changes on health utility. The changes in Usual Activity and Pain/discomfort dimensions were the main driver for the change in health states. Among the 15 patients who reported improved health states, 10 of them improved in the usual activity dimension and 7 improved in the pain/discomfort dimension. Among the 19 patients who reported worsening health states, 10 of them got worse in the usual activity dimension and 9 in the pain/discomfort dimension.

Table F2 shows the descriptive statistics of changes in health utility between baseline and follow-up for adult patients and by diagnostic group and value aspects. Using a US value set instead of a English value set did not significantly change the results.

| Table F2 Sensitivity analysis of responsiveness of EQ-5D-5L between baseline and follow-up of adult CND patients for the full sample and by diagnostic outcome and value aspects | | | | | | | |
| --- | --- | --- | --- | --- | --- | --- | --- |
|  |  | English value set | | US value set | | AU value set | |
|  | N | Mean | SD | Mean | SD | Mean | SD |
| Full sample | 61 | -0.01 | 0.12 | -0.01 | 0.15 | -0.004 | 0.13 |
| By diagnostic group |  |  |  |  |  |  |  |
| Diagnosis | 10 | -0.05 | 0.15 | -0.07 | 0.17 | -0.06 | 0.16 |
| No diagnosis | 51 | 0.00 | 0.12 | 0.00 | 0.15 | 0.01 | 0.12 |
| By value aspects |  |  |  |  |  |  |  |
| Value in information | |  |  |  |  |  |  |
| Yes | 37 | -0.02 | 0.13 | -0.03 | 0.15 | -0.02 | 0.13 |
| No | 17 | 0.01 | 0.12 | 0.02 | 0.17 | 0.01 | 0.12 |
| Value in chance to improve health | | |  |  |  |  |  |
| Yes | 42 | 0.00 | 0.14 | -0.01 | 0.17 | -0.005 | 0.14 |
| No | 12 | -0.02 | 0.09 | -0.02 | 0.11 | -0.02 | 0.09 |

It is noteworthy that the standard deviations of the estimated mean change in health utility among the full sample were high, indicating a number of respondents experienced an increase in health utility. We further examined the change of EQ-5D-5L profiles of those who experienced increased utility. As shown in Table F3, there were 23 adult patients had an increase in health utility after genomic testing, 9 of which had improvements on anxiety/depression dimension and 8 remained “no problem” on this dimension. As shown in Table F4, there were 28 adult patients who had a decline in health utility, including 10 had mixed changes on health states and 18 had worsen health state on at least one dimension while no improvements on others. Of these 28 adult patients, 18 (64%) had improvements or no change on anxiety/depression dimension, indicating the declines in health utilities were largely driven by declines in physical health.

| Table F3 Health states profile before and after genomic testing among patients had increased EQ-5D-5L utilities | | | | | | |
| --- | --- | --- | --- | --- | --- | --- |
| phenotype | Diagnostic Outcomes | Baseline | Follow up | Change in health utilities | PCHC | Changes on anxiety/ depression |
| Ataxia | 0 | 43434 | 43423 | 0.209 | Improved | Improved |
| Ataxia | 0 | 51551 | 51122 | 0.371 | Mixed | Worsen |
| Ataxia | 0 | 32211 | 21111 | 0.058 | Improved | No problem No change |
| Ataxia | 0 | 43522 | 43421 | 0.185 | Improved | Improved |
| Ataxia | 0 | 44522 | 43422 | 0.308 | Improved | No change |
| Ataxia | 0 | 32331 | 32221 | 0.092 | Improved | No problem No change |
| Ataxia | 0 | 11114 | 21223 | 0.089 | Mixed | Improved |
| Complex NOS | 0 | 31132 | 21113 | 0.075 | Mixed | Worsen |
| Complex NOS | 0 | 11121 | 11111 | 0.044 | Improved | No problem No change |
| Complex NOS | 0 | 44553 | 44452 | 0.034 | Improved | Improved |
| Complex NOS | 0 | 22231 | 32221 | 0.009 | Mixed | No problem No change |
| Complex NOS | 0 | 44554 | 43453 | 0.327 | Improved | Improved |
| Dystonia | 0 | 11121 | 21111 | 0.005 | Mixed | No problem No change |
| Dystonia | 0 | 41442 | 41342 | 0.107 | Improved | No change |
| HSP | 0 | 31312 | 32212 | 0.025 | Mixed | No change |
| HSP | 0 | 31233 | 31321 | 0.048 | Mixed | Improved |
| PD | 0 | 11131 | 11121 | 0.037 | Improved | No problem No change |
| PD | 0 | 22232 | 32231 | 0.004 | Mixed | Improved |
| PD | 0 | 32332 | 11221 | 0.221 | Improved | Improved |
| PD | 0 | 11121 | 11111 | 0.044 | Improved | No problem No change |
| PD | 0 | 21221 | 21111 | 0.044 | Improved | No problem No change |
| PD | 1 | 32214 | 32212 | 0.206 | Improved | Improved |
| Spastic Paraplegia | 1 | 21322 | 21222 | 0.055 | Improved | No change |

| Table F4 Health states profile before and after genomic testing among patients had declined EQ-5D-5L utilities | | | | | | |
| --- | --- | --- | --- | --- | --- | --- |
| phenotype | Diagnostic Outcomes | Baseline | Follow up | Change in health utilities | PCHC | Changes on anxiety/ depression |
| Ataxia | 0 | 32332 | 32432 | -0.107 | Worsen | No change |
| Ataxia | 0 | 22321 | 31422 | -0.137 | Mixed | Worsen |
| Ataxia | 0 | 31331 | 41231 | -0.115 | Mixed | No problem No change |
| Complex NOS | 0 | 21343 | 32342 | -0.024 | Mixed | Improved |
| Complex NOS | 0 | 44434 | 44435 | -0.153 | Worsen | Worsen |
| Complex NOS | 0 | 21325 | 22435 | -0.174 | Worsen | No change |
| Dementia | 0 | 11111 | 32312 | -0.184 | Worsen | Worsen |
| Dementia | 0 | 11121 | 11223 | -0.066 | Worsen | Worsen |
| Dementia | 0 | 11123 | 11132 | -0.003 | Mixed | Improved |
| Dystonia | 0 | 11122 | 21221 | -0.007 | Mixed | Improved |
| Dystonia | 0 | 11111 | 11122 | -0.076 | Worsen | Worsen |
| Dystonia | 0 | 11112 | 11122 | -0.044 | Worsen | No change |
| HSP | 0 | 32211 | 33311 | -0.083 | Worsen | No problem No change |
| HSP | 0 | 32222 | 32322 | -0.055 | Worsen | No change |
| HSP | 0 | 52221 | 52231 | -0.037 | Worsen | No problem No change |
| HSP | 0 | 41241 | 41342 | -0.087 | Worsen | Worsen |
| HSP | 0 | 32333 | 42332 | -0.136 | Mixed | Improved |
| HSP | 0 | 41321 | 42421 | -0.137 | Worsen | No problem No change |
| MND | 0 | 13321 | 13322 | -0.032 | Worsen | Worsen |
| PD | 0 | 33343 | 44433 | -0.237 | Mixed | No change |
| PD | 0 | 11121 | 21121 | -0.039 | Worsen | No problem No change |
| PD | 0 | 22231 | 23232 | -0.06 | Worsen | Worsen |
| PD | 0 | 22231 | 22232 | -0.032 | Worsen | Worsen |
| MND | 1 | 53311 | 52321 | -0.016 | Mixed | No problem No change |
| Spastic Paraplegia | 1 | 53313 | 55512 | -0.236 | Mixed | Improved |
| Spastic Paraplegia | 1 | 32321 | 43432 | -0.374 | Worsen | Worsen |
| Spastic Paraplegia | 1 | 21222 | 31412 | -0.146 | Mixed | No change |
| Spastic Paraplegia | 1 | 41442 | 43442 | -0.058 | Worsen | No change |

Appendix G Additional analyses on changes in health outcomes before and after genomic sequencing among dilated cardiomyopathy (DCM) patients

We assessed the changes in AQoL-8D utility scores and dimensions. The AQoL-8D is a validated measure that contains 35 items and comprises eight separately scored dimensions, consisting of independent living, relationships, mental health, coping, pain, senses, self-worth, and life satisfaction. [[25](#_ENREF_25)]

Table G1 shows the changes in AQoL-8D dimension scores among DCM patients who had increased AQoL-8D utility. Among the 14 patients who reported improvements in health utility, 13 (93%) had improvements in the Relationship and Self-Worth dimensions, and 12 (86%) improved in the Happiness dimension.

| Table G1 Change in AQoL dimensions before and after genomic testing among patients had increased AQoL utilities | | | | | | | | | | |
| --- | --- | --- | --- | --- | --- | --- | --- | --- | --- | --- |
| Diagnostic Outcomes | PCHC | Changes in health utility scores and dimension scores | | | | | | | | |
|  |  | Utility scores | Independent living | Happiness | Mental Health | Coping | Relationships | Self-Worth | Pain | Senses |
| Confirmed | mixed | 0.06 | 0.00 | 0.00 | 0.00 | 0.02 | 0.15 | 0.05 | -0.08 | 0.10 |
| Confirmed | mixed | 0.13 | 0.00 | 0.06 | 0.17 | -0.05 | 0.01 | 0.07 | 0.25 | 0.00 |
| Confirmed | mixed | 0.10 | 0.00 | 0.18 | 0.09 | 0.00 | 0.14 | 0.00 | 0.00 | -0.03 |
| Unconfirmed | mixed | 0.08 | -0.04 | 0.00 | 0.00 | 0.00 | 0.18 | 0.16 | 0.10 | -0.11 |
| Unconfirmed | Improved | 0.13 | 0.15 | 0.03 | 0.31 | 0.11 | 0.14 | 0.00 | 0.00 | 0.00 |
| Unconfirmed | mixed | 0.02 | 0.01 | 0.11 | -0.06 | 0.16 | -0.04 | 0.14 | -0.09 | -0.16 |
| Unconfirmed | mixed | 0.06 | 0.03 | 0.00 | 0.06 | 0.00 | 0.09 | -0.02 | 0.06 | 0.00 |
| Unconfirmed | mixed | 0.16 | 0.00 | 0.05 | 0.16 | 0.10 | 0.14 | 0.16 | -0.07 | 0.00 |
| Unconfirmed | mixed | 0.15 | 0.03 | 0.19 | -0.03 | 0.10 | 0.18 | 0.07 | 0.00 | 0.15 |
| Unconfirmed | mixed | 0.16 | 0.29 | -0.03 | -0.02 | 0.09 | 0.04 | 0.18 | 0.39 | -0.10 |
| Unconfirmed | mixed | 0.04 | 0.07 | -0.01 | -0.07 | -0.20 | 0.00 | 0.08 | 0.50 | 0.05 |
| Unconfirmed | mixed | 0.12 | 0.14 | 0.19 | 0.12 | 0.25 | 0.11 | 0.00 | -0.37 | 0.00 |
| Unconfirmed | mixed | 0.18 | 0.10 | 0.00 | 0.04 | 0.04 | 0.13 | 0.38 | -0.06 | 0.40 |
| Unconfirmed | Improved | 0.07 | 0.04 | 0.06 | 0.03 | 0.00 | 0.07 | 0.06 | 0.08 | 0.00 |

Table G2 shows the changes in AQoL-8D dimension scores among 19 DCM patients who had increased AQoL-8D utility. They experienced worsened health states in most physical and psychosocial domains.

| Table G2 Change in AQoL dimensions before and after genomic testing among patients had declined AQoL utilities | | | | | | | | | | |
| --- | --- | --- | --- | --- | --- | --- | --- | --- | --- | --- |
| Diagnostic Outcomes | PCHC | Changes in health utility scores and dimension scores | | | | | | | | |
|  |  | Utility scores | Independent living | Happiness | Mental Health | Coping | Relationships | Self-Worth | Pain | Senses |
| Unconfirmed | mixed | -0.02 | 0.03 | 0.00 | -0.04 | 0.00 | 0.00 | 0.01 | 0.00 | -0.08 |
| Unconfirmed | mixed | -0.08 | 0.00 | -0.07 | -0.13 | -0.02 | 0.01 | 0.00 | 0.00 | -0.09 |
| Unconfirmed | mixed | -0.03 | 0.21 | -0.04 | -0.14 | 0.07 | -0.07 | 0.00 | 0.00 | 0.00 |
| Unconfirmed | mixed | 0.00 | 0.00 | 0.00 | -0.03 | 0.00 | 0.00 | 0.05 | 0.00 | -0.03 |
| Unconfirmed | worsen | -0.26 | 0.00 | -0.27 | -0.12 | -0.25 | -0.11 | -0.11 | -0.15 | -0.15 |
| Unconfirmed | mixed | -0.02 | 0.00 | 0.00 | -0.08 | -0.10 | 0.00 | 0.00 | -0.05 | 0.15 |
| Unconfirmed | mixed | -0.02 | 0.09 | -0.04 | 0.01 | -0.03 | -0.02 | 0.00 | -0.06 | 0.00 |
| Unconfirmed | mixed | -0.07 | 0.02 | -0.01 | -0.12 | -0.03 | -0.04 | -0.08 | 0.05 | -0.09 |
| Unconfirmed | mixed | -0.03 | -0.30 | -0.04 | 0.15 | 0.03 | -0.07 | 0.00 | 0.00 | -0.10 |
| Unconfirmed | mixed | -0.04 | -0.07 | -0.06 | -0.06 | -0.10 | 0.12 | -0.06 | -0.11 | 0.11 |
| Unconfirmed | mixed | -0.17 | -0.03 | -0.12 | -0.15 | -0.21 | -0.18 | 0.02 | 0.00 | 0.00 |
| Unconfirmed | mixed | -0.09 | 0.03 | -0.04 | 0.00 | -0.16 | 0.01 | -0.04 | -0.13 | -0.10 |
| Unconfirmed | mixed | -0.09 | -0.24 | 0.00 | -0.15 | 0.00 | -0.07 | -0.06 | 0.15 | 0.00 |
| Unconfirmed | mixed | 0.00 | 0.09 | 0.00 | 0.01 | 0.10 | -0.08 | 0.11 | -0.27 | 0.11 |
| Unconfirmed | mixed | -0.08 | 0.00 | 0.00 | 0.00 | 0.11 | -0.07 | -0.06 | -0.23 | -0.09 |
| Unconfirmed | mixed | 0.00 | 0.00 | 0.04 | -0.07 | 0.00 | 0.00 | -0.03 | 0.05 | 0.00 |
| Confirmed | mixed | -0.02 | 0.00 | 0.00 | -0.01 | 0.07 | -0.05 | -0.06 | -0.05 | 0.00 |
| Confirmed | mixed | -0.03 | -0.05 | -0.05 | 0.17 | 0.00 | 0.00 | -0.14 | -0.03 | -0.10 |
| Confirmed | mixed | -0.04 | -0.07 | 0.00 | -0.12 | -0.11 | 0.05 | 0.05 | -0.17 | 0.17 |

Appendix H Change in patient-reported outcomes by each level of each value aspects of genomic sequencing

| **Table H1 Mean change in health utility by factor-based scores for each value aspect among NDs parents** | | | | | | | | | | | | | | | | | | |
| --- | --- | --- | --- | --- | --- | --- | --- | --- | --- | --- | --- | --- | --- | --- | --- | --- | --- | --- |
|  | **Value in information** | | | | | | | | | | | |  |  |  |  |  |  |
| Factor-based scores | 0 | | | 1 | | | 2 | | | 3 | | | 4 | | | 5 | | |
|  | N | Mean | SD | N | Mean | SD | N | Mean | SD | N | Mean | SD | N | Mean | SD | N | Mean | SD |
| Neurodevelopmental disorders (parents) | |  |  |  |  |  |  |  |  |  |  |  |  |  |  |  |  |  |
| SF-6D utility scores | 13 | -0.04 | 0.13 | 7 | -0.09 | 0.13 | 9 | -0.03 | 0.13 | 6 | -0.01 | 0.15 | 7 | 0.04 | 0.10 | 15 | -0.04 | 0.09 |
| PCS score | 13 | -2.24 | 8.25 | 7 | -2.75 | 11.90 | 9 | -4.51 | 14.72 | 6 | 2.37 | 6.94 | 7 | -3.36 | 10.06 | 15 | -1.78 | 9.51 |
| MCS score | 13 | -5.26 | 14.63 | 7 | -5.02 | 13.59 | 9 | 4.78 | 17.44 | 6 | -3.33 | 6.91 | 7 | 7.84 | 9.03 | 15 | -0.72 | 13.20 |
| Note: For NDs parents, a higher SF-6D score, SF-12 PCS score and SF-12 MCS score indicate better health. | | | | | | | | | | | | | | | | | | |

| **Table H2 Mean change in health utility by factor based scores for each value aspect in GKDs, CNDs and DCM cohort** | | | | | | | | | | | | | | | | | | | | | |
| --- | --- | --- | --- | --- | --- | --- | --- | --- | --- | --- | --- | --- | --- | --- | --- | --- | --- | --- | --- | --- | --- |
|  | **Value in information** | | | | | | | | | | | | **Value in chance to improve health** | | | | | | | | |
| Factor-based scores | 0 | | | 1 | | | 2 | | | 3 | | | 0 | | | 1 | | | 2 | | |
|  | N | Mean | SD | N | Mean | SD | N | Mean | SD | N | Mean | SD | N | Mean | SD | N | Mean | SD | N | Mean | SD |
| Genetic kidney disease (adult patients) | | | | | | | | | | | | | | | | | | | | | |
| SF-6D utility scores | 12 | -0.01 | 0.10 | 8 | -0.02 | 0.08 | 9 | 0.00 | 0.08 | 38 | -0.01 | 0.10 | 12 | 0.00 | 0.12 | 20 | 0.00 | 0.08 | 35 | -0.02 | 0.09 |
| PCS score | 12 | 2.32 | 6.93 | 8 | -1.10 | 9.84 | 9 | -0.20 | 8.32 | 37 | 0.64 | 8.61 | 12 | -3.06 | 10.20 | 19 | 0.46 | 6.89 | 35 | 1.97 | 8.17 |
| MCS score | 12 | -0.78 | 9.97 | 8 | 1.14 | 7.69 | 9 | 0.62 | 5.71 | 37 | -1.47 | 8.51 | 12 | 3.52 | 9.97 | 19 | 0.68 | 6.25 | 35 | -2.97 | 8.07 |
| Genetic kidney disease (parents) | | | | | | | | | | | | | | | | | | | | | |
| SF-6D utility scores | 2 | 0.00 | 0.06 | 1 | 0.12 | . | 3 | 0.09 | 0.18 | 13 | -0.04 | 0.06 | 1 | 0.05 | . | 4 | -0.02 | 0.11 | 14 | -0.01 | 0.10 |
| PCS score | 2 | 1.61 | 5.74 | 1 | 5.61 | . | 3 | 1.64 | 1.97 | 13 | -1.78 | 7.95 | 1 | 5.66 | . | 4 | 0.82 | 4.19 | 14 | -1.31 | 7.71 |
| MCS score | 2 | 0.22 | 5.70 | 1 | 11.28 | . | 3 | 5.59 | 6.89 | 13 | -3.22 | 9.25 | 1 | 4.25 | . | 4 | 0.42 | 8.18 | 14 | -1.38 | 9.86 |
| Complex Neurological Disorders (adult patients) | | | | | | | | | | | | | | | | | | | | | |
| EQ-5D-5L utilities | 17 | 0.01 | 0.12 | 9 | -0.08 | 0.13 | 4 | -0.002 | 0.11 | 24 | 0.02 | 0.15 | 12 | -0.03 | 0.07 | 12 | 0.00 | 0.17 | 30 | 0.01 | 0.14 |
| VAS score | 17 | 0.03 | 0.14 | 8 | -0.02 | 0.24 | 4 | -0.12 | 0.18 | 23 | -0.01 | 0.18 | 12 | -0.04 | 0.13 | 11 | -0.01 | 0.17 | 29 | 0.01 | 0.20 |
| Depression z-score | 15 | -0.33 | 0.65 | 7 | 0.12 | 0.49 | 4 | 0.22 | 0.59 | 19 | 0.24 | 1.93 | 12 | 0.00 | 0.89 | 10 | 0.06 | 0.67 | 23 | 0.09 | 1.72 |
| Anxiety z-score | 15 | -0.03 | 0.99 | 7 | -0.06 | 0.98 | 4 | 0.71 | 0.39 | 19 | 0.32 | 1.46 | 12 | 0.04 | 0.97 | 10 | 0.29 | 0.88 | 23 | -0.21 | 1.40 |
| Stress z-score | 15 | 0.20 | 0.52 | 7 | -0.18 | 0.82 | 4 | 0.19 | 0.60 | 19 | 0.09 | 1.45 | 12 | 0.27 | 0.61 | 10 | 0.10 | 0.75 | 23 | 0.00 | 1.31 |
| NeuroQoL T score | 15 | -0.19 | 4.79 | 8 | -0.69 | 1.85 | 4 | -5.78 | 6.92 | 23 | -0.47 | 5.78 | 11 | 0.35 | 5.01 | 11 | -1.89 | 5.52 | 28 | -0.90 | 5.26 |
| Dilated cardiomyopathy | | | | | | | | | | | | | | | | | | | | | |
| AQoL utility score | 11 | 0.02 | 0.11 | 0 |  |  | 2 | -0.02 | 0.00 | 15 | 0.01 | 0.10 | 5 | 0.00 | 0.16 | 5 | -0.01 | 0.06 | 18 | 0.02 | 0.10 |
| Physical | 11 | 0.04 | 0.16 | 0 |  |  | 2 | -0.14 | 0.14 | 15 | -0.01 | 0.10 | 5 | -0.05 | 0.16 | 5 | -0.02 | 0.08 | 18 | 0.02 | 0.14 |
| Psychosocial | 11 | 0.01 | 0.09 | 0 |  |  | 2 | 0.02 | 0.05 | 15 | 0.00 | 0.10 | 5 | 0.02 | 0.11 | 5 | -0.01 | 0.05 | 18 | 0.01 | 0.10 |
| Note: For GKDs parents and GKDs adults, a higher SF-6D score, SF-12 PCS score and SF-12 MCS score indicate better health. For CNDs adult patients, a higher EQ-5D utility score and VAS score indicates better health. We rescaled VAS score to a 0-1 scale; DASS scale score was converted to z-score to enable comparison between DASS scale scores. We rescaled the DASS scores so that higher z-score indicates better health state. A higher Neuro-QoL T-score represents more of the concept being measured. For DCM adult patients, a higher AQoL utility score and the two component scores indicate better health. | | | | | | | | | | | | | | | | | | | | | |

| Table H3 Mean change in health utility by each value statement | | | | | | | | | | | | | | | | |
| --- | --- | --- | --- | --- | --- | --- | --- | --- | --- | --- | --- | --- | --- | --- | --- | --- |
|  |  | NDs parents | | | GKDs adults | | | GKDs parents | | | CNDs | | | DCM | | |
|  | Value aspects | Yes | No | NA | Yes | No | NA | Yes | No | NA | Yes | No | NA | Yes | No | NA |
| Q1 | Ongoing investigations no longer necessary | -0.02 | -0.05 | -0.01 | -0.01 | -0.02 | 0.01 | -0.04 | 0.16 | -0.02 | -0.0004 | -0.02 | -0.003 | 0.01 | 0.01 | 0.02 |
| Q2 | Knowing the cause / explanation for the condition | -0.03 | -0.04 | -0.02 | -0.01 | -0.06 | 0.02 | -0.03 | 0.04 | 0.01 | 0.01 | -0.04 | 0.002 | 0.00 | 0.05 | 0.02 |
| Q3 | Information for my own family planning | -0.03 | -0.04 | -0.01 | -0.01 | -0.02 | 0.00 | -0.04 | -0.01 | 0.01 | 0.005 | -0.01 | -0.01 | 0.03 | 0.01 | -0.01 |
| Q4 | Information for other members of my family | -0.02 | -0.05 | -0.03 | 0.00 | -0.05 | 0.02 | -0.02 | 0.06 | -0.02 | -0.01 | -0.02 | 0.01 | 0.01 | 0.08 | 0.02 |
| Q5 | Information for treatment / management of the condition | -0.01 | -0.06 | -0.02 | -0.01 | -0.03 | 0.02 | -0.01 | 0.00 | -0.01 | 0.01 | -0.02 | -0.01 | 0.00 | 0.06 | 0.01 |
| Q6 | Information regarding prognosis/ knowing what to expect in the future | -0.03 | -0.05 | -0.01 | -0.01 | 0.01 | 0.00 | -0.02 | 0.04 | -0.04 | -0.004 | 0.002 | -0.01 | 0.00 | 0.07 | 0.01 |
| Q7 | Have had access to the most recent advances in medicine | NA | NA | NA | -0.02 | -0.02 | 0.03 | -0.01 | 0.08 | -0.06 | 0.00 | -0.04 | 0.01 | 0.01 | 0.01 | 0.01 |
| Q8 | I have done everything I can to improve health | NA | NA | NA | -0.01 | 0.00 | 0.01 | -0.01 | -0.01 | -0.04 | 0.01 | -0.03 | -0.03 | 0.01 | 0.11 | 0.00 |
| Q9 | Ability to connect with others with the same condition | -0.05 | -0.04 | -0.01 | -0.02 | -0.02 | 0.01 | -0.04 | -0.01 | 0.02 | 0.02 | -0.04 | -0.004 | 0.00 | 0.01 | 0.02 |
| Q10 | To contribute to research | -0.03 | -0.05 | -0.03 | NA | NA | NA | NA | NA | NA | NA | NA | NA | 0.00 | 0.15 | 0.03 |
| Q11 | Data be examined in more detail to find answer | NA | NA | NA | NA | NA | NA | NA | NA | NA | NA | NA | NA | 0.00 | 0.00 | 0.06 |
| Yes if a respondent answered “extremely valuable” or “valuable”, No if a respondent answered “neutral”, “not valuable”. NA refers to those who chose not applicable. | | | | | | | | | | | | | | | | |

Appendix I Change in health utilities when controlling baseline physical health among parents of children affected by NDs and adult GKD patients and parents

| Table I1 Change in health utilities when holding baseline physical health by diagnostic outcome | | | | | | |
| --- | --- | --- | --- | --- | --- | --- |
|  | Diagnosis | | | No diagnosis | | |
|  | Change | | | Change | | |
| Changes in health utilities | N | Mean | SD | N | Mean | SD |
| NDs (parents) |  |  |  |  |  |  |
| Change in SF-6D | 20 | -0.01 | 0.08 | 42 | -0.04 | 0.14 |
| Adjusted change in SF-6D | 20 | -0.02 | 0.07 | 42 | -0.02 | 0.07 |
| GKD (adult patients) |  |  |  |  |  |  |
| Change in SF-6D | 40 | -0.02 | 0.09 | 36 | 0.01 | 0.08 |
| Adjusted change in SF-6D | 40 | -0.02 | 0.07 | 36 | 0.00 | 0.04 |
| GKD (parents) |  |  |  |  |  |  |
| Change in SF-6D | 11 | -0.04 | 0.07 | 11 | 0.01 | 0.11 |
| Adjusted change in SF-6D | 11 | -0.02 | 0.06 | 11 | 0.00 | 0.07 |

| Table I3 Change in health utilities when holding baseline physical health by clinical usefulness | | | | | | | | | | | | |
| --- | --- | --- | --- | --- | --- | --- | --- | --- | --- | --- | --- | --- |
|  | NGS useful in changing management | | | | | | NGS useful in improving health | | | | | |
|  | Yes | | | No |  |  | Yes |  |  | No |  |  |
| Changes in health utilities | N | Mean | SD | N | Mean | SD | N | Mean | SD | N | Mean | SD |
| NDs (parents) |  |  |  |  |  |  |  |  |  |  |  |  |
| change in SF-6D | 13 | -0.04 | 0.07 | 9 | -0.02 | 0.13 | 13 | -0.02 | 0.05 | 9 | -0.05 | 0.15 |
| Adjusted change in SF-6D | 13 | -0.03 | 0.06 | 9 | 0.00 | 0.07 | 13 | -0.02 | 0.06 | 9 | -0.01 | 0.08 |

| Table I2 Change in health utilities when holding baseline physical health by personal utility | | | | | | | | | | | | |
| --- | --- | --- | --- | --- | --- | --- | --- | --- | --- | --- | --- | --- |
|  | Value in information | | | | | | Value in chance to improve health | | | | | |
|  | Yes | | | No | | | Yes | | | No | | |
| Changes in health utilities | N | Mean | SD | N | Mean | SD | N | Mean | SD | N | Mean | SD |
| NDs (parents) |  |  |  |  |  |  |  |  |  |  |  |  |
| Change in SF-6D | 44 | -0.03 | 0.12 | 13 | -0.04 | 0.13 |  |  |  |  |  |  |
| Adjusted change in SF-6D | 44 | -0.02 | 0.06 | 13 | -0.02 | 0.07 |  |  |  |  |  |  |
| GKD (adult patients) |  |  |  |  |  |  |  |  |  |  |  |  |
| Change in SF-6D | 55 | -0.01 | 0.09 | 12 | -0.01 | 0.10 | 55 | -0.01 | 0.09 | 12 | 0.00 | 0.12 |
| Adjusted change in SF-6D | 55 | -0.01 | 0.07 | 12 | -0.01 | 0.04 | 55 | -0.01 | 0.06 | 12 | 0.01 | 0.07 |
| GKD (parents) |  |  |  |  |  |  |  |  |  |  |  |  |
| Change in SF-6D | 17 | -0.01 | 0.10 | 2 | 0.00 | 0.06 | 18 | -0.01 | 0.10 | 1 | 0.05 |  |
| Adjusted change in SF-6D | 17 | 0.00 | 0.07 | 2 | 0.02 | 0.03 | 18 | 0.00 | 0.06 | 1 | 0.05 |  |

Reference

1. Stark Z, Boughtwood T, Phillips P, Christodoulou J, Hansen DP, Braithwaite J, Newson AJ, Gaff CL, Sinclair AH, North KN: **Australian Genomics: A Federated Model for Integrating Genomics into Healthcare.** *The American Journal of Human Genetics* 2019, **105:**7-14.

2. Taylor N, Best S, Martyn M, Long JC, North KN, Braithwaite J, Gaff C: **A transformative translational change programme to introduce genomics into healthcare: a complexity and implementation science study protocol.** *BMJ Open* 2019, **9:**e024681.

3. Jayasinghe K, Stark Z, Kerr PG, Gaff C, Martyn M, Whitlam J, Creighton B, Donaldson E, Hunter M, Jarmolowicz A, et al: **Clinical impact of genomic testing in patients with suspected monogenic kidney disease.** *Genet Med* 2021, **23:**183-191.

4. Jayasinghe K, Stark Z, Patel C, Mallawaarachchi A, McCarthy H, Faull R, Chakera A, Sundaram M, Jose M, Kerr P, et al: **Comprehensive evaluation of a prospective Australian patient cohort with suspected genetic kidney disease undergoing clinical genomic testing: a study protocol.** *BMJ Open* 2019, **9:**e029541.

5. Eratne D, Schneider A, Lynch E, Martyn M, Velakoulis D, Fahey M, Kwan P, Leventer R, Rafehi H, Chong B, et al: **The clinical utility of exome sequencing and extended bioinformatic analyses in adolescents and adults with a broad range of neurological phenotypes: an Australian perspective.** *Journal of the Neurological Sciences* 2021, **420:**117260.

6. Ramchand J, Wallis M, Macciocca I, Lynch E, Farouque O, Martyn M, Phelan D, Chong B, Lockwood S, Weintraub R, et al: **Prospective Evaluation of the Utility of Whole Exome Sequencing in Dilated Cardiomyopathy.** *Journal of the American Heart Association* 2020, **9:**e013346.

7. Catchpool M, Ramchand J, Martyn M, Hare DL, James PA, Trainer AH, Knight J, Goranitis I: **A cost-effectiveness model of genetic testing and periodical clinical screening for the evaluation of families with dilated cardiomyopathy.** *Genetics in Medicine* 2019, **21:**2815-2822.

8. Ramchand J, Wallis M, Macciocca I, Lynch E, Farouque O, Martyn M, Phelan D, Chong B, Lockwood S, Weintraub R, et al: **Prospective Evaluation of the Utility of Whole Exome Sequencing in Dilated Cardiomyopathy.** *Journal of the American Heart Association* 2020, **9:**e013346.

9. Meienberg J, Bruggmann R, Oexle K, Matyas G: **Clinical sequencing: is WGS the better WES?** *Hum Genet* 2016, **135:**359-362.

10. Lee I-H, Lin Y, Alvarez WJ, Hernandez-Ferrer C, Mandl KD, Kong SW: **WEScover: selection between clinical whole exome sequencing and gene panel testing.** *BMC Bioinformatics* 2021, **22:**259.

11. **WES vs. WGS vs. Custom Panels** [<https://sequencing.roche.com/us/en/article-listing/wes-wgs-custom.html>]

12. Rencher AC, William FC: *Methods of Multivariate Analysis.* John Wiley & Sons, Incorporated; 2012.

13. Gallin JI, Ognibene FP, Johnson LL: *Principles and Practice of Clinical Research.* Boston: Academic Press; 2018.

14. Kim JO, Mueller CW: *introduction to factor analysis. What it is and how to do it. .* CA: Sage.: housand Oaks; 1978.

15. Osborne JW: **"What is Rotating in Exploratory Factor Analysis?,"** *Practical Assessment, Research, and Evaluation* 2019, **20**.

16. DiStefano C, Zhu M, Mîndrilã D: **Understanding and Using Factor Scores: Considerations for the Applied Researcher.** *Practical Assessment, Research, and Evaluation* 2009, **14:**Article 20.

17. Calvert MJ, Cruz Rivera S, Retzer A, Hughes SE, Campbell L, Molony-Oates B, Aiyegbusi OL, Stover AM, Wilson R, McMullan C, et al: **Patient reported outcome assessment must be inclusive and equitable.** *Nature Medicine* 2022, **28:**1120-1124.

18. Ratnayake I, Ahern S, Ruseckaite R: **A systematic review of patient-reported outcome measures (PROMs) in cystic fibrosis.** *BMJ Open* 2020, **10:**e033867.

19. Catchpool M, Gold L, Grobler AC, Clifford SA, Wake M: **Health-related quality of life: population epidemiology and concordance in Australian children aged 11–12 years and their parents.** *BMJ Open* 2019, **9:**157.

20. Norman R, Church J, van den Berg B, Goodall S: **Australian health-related quality of life population norms derived from the SF-6D.** *Australian and New Zealand Journal of Public Health* 2013, **37:**17-23.

21. McCaffrey N, Kaambwa B, Currow DC, Ratcliffe J: **Health-related quality of life measured using the EQ-5D–5L: South Australian population norms.** *Health and Quality of Life Outcomes* 2016, **14:**133.

22. Maxwell A, Özmen M, Iezzi A, J. R: **Deriving population norms for the AQoL-6D and AQoL-8D multi-attribute utility instruments from web-based data. .** *Quality of Life Research* 2016, **doi: 10.1007/s11136-016-1337-z**.

23. Brazier JE, Roberts J: **The estimation of a preference-based measure of health from the SF-12.** *Medical care* 2004**:**851-859.

24. Devlin N, Parkin D, Browne J: **Using the EQ-5D as a performance measurement tool in the NHS.** *Health Economics* 2010, **19:**886-905.

25. Catchpool M, Ramchand J, Hare DL, Martyn M, Goranitis I: **Mapping the Minnesota Living with Heart Failure Questionnaire (MLHFQ) onto the Assessment of Quality of Life 8D (AQoL-8D) utility scores.** *Quality of Life Research* 2020, **29:**2815-2822.
